# Supplementary material for: Perceptions of virtual primary care physicians: A focus group study of medical and data science graduate students
Source: PLoS One. 2020 Dec 17;15(12):e0243641. doi: 10.1371/journal.pone.0243641 (PMC7745971; doi:10.1371/journal.pone.0243641)
Supplement: S1 Appendix — (DOCX) [file pone.0243641.s001.docx]

S1 Appendix – Focus group questions

**Moderator Instructions**

These questions should be modified as needed to maintain the natural flow of the conversation and to explore topics which arise in the course of the focus group. The script below is a general guide to direct the conversation. Probes should be revised as needed to encourage elaboration on answers and to maintain the flow of the conversation. If a participant goes off topic, but is providing useful content, continue probing as needed then redirect to the original script. If off-topic conversation does not appear relevant, a casual redirect to original script should be made. Notes of most salient points should be kept by the recorder during interviews. Notes will be used to help PIs while awaiting transcriptions.

1. What is your view of using a virtual PCP?
2. What advantages do you see?

- Probe: Are there any situations in which you would prefer a vPCP?

1. What are some drawbacks?
   - Probe: Any situations where you would NOT be willing to use a vPCP?
2. How do you envision using a vPCP
   - Probes: biometrics/monitoring vitals, patient education, patient engagement

1. Could this be a reality?
