# Supplementary material for: Perceptions of virtual primary care physicians: A focus group study of medical and data science graduate students
Source: PLoS One. 2020 Dec 17;15(12):e0243641. doi: 10.1371/journal.pone.0243641 (PMC7745971; doi:10.1371/journal.pone.0243641)
Supplement: S1 File — (ZIP) [file pone.0243641.s002.zip › Anonymized Transcripts/vPCP Focus Group 1 anonymized.docx]

vPCP Focus Group 1

March 29, 2019

Present:

Moderator

Two note-takers

Five 1^st^ year med students

Moderator: So, the first question is, in general, what is your view of using a virtual primary care provider?

5: I think it has a lot of potential regarding the ability to screen patients beforehand, figure out where are their preliminary problems, and figure out at that point what are the next steps because already there’s—we think in kind of algorithms. In that, as medical students, we think about what [what is this patient bringing, and—let me pause for a second. You’re asking me to think about it as a patient. I’m sorry.

Moderator: Right. Good.

5: My first response as a patient would be maybe I should look it up on the internet, and that’s already a first step of looking up what I have, what I think is important, and seeing what Google tells me. So, I don’t see it as a—I think a comprehensive response to what I would expect my provider to do, but I think it’s a good gateway for me to figure out what I can bring to the table.

Moderator: Okay. Yeah. Other thoughts?

4: From my perspective, I was ill last semester, and I had an upper respiratory infection. And it would have been really helpful for me not to have to drive to a clinic or someone to tell me that I had an upper respiratory infection because I had coughing, sneezing, runny nose, headache, things like that. So, I think applications of diagnosing patients and prescribing them over the counter medications, saying drink some fluids, get some rest, call me back in a week, this kind of thing really saved me, personally as a patient, a lot of hassle.

Moderator: Okay. So, you see it a convenience.

4: Exactly. So, in terms of convenience, much more convenient, especially being as busy as I am as a student. I don’t wanna waste the time to go wait in the waiting room, and you know, have to do that whereas I could just sit home in my pajamas and talk to someone 5 minutes—in and out.

Moderator: Mmm hmm. Alright. Any new thoughts?

1: I think, my knee-jerk reaction is, like, absolutely not. And I think the more I kind of reflect on it, it would be very situation-specific whether or not I would consider use of virtual PCP. If I—and see I feel like I see flaws in that already. So, if I feel like I have a headache due to the stress that I’ve had at work for the last week, I might get prescribed some kind of medication for a headache, when really, I have a rapidly growing brain tumor. And only people are going to be able to search and kind of differentiate that gray area, and I worry that something like that is going to miss that. And furthermore, if it misses it, right now we live in a litigation nation that centers on people and companies, who’s gonna be at fault? Is it gonna be the algorithm? Is it gonna be the person that designed the algorithm? Like, when something goes awry because something will go awry eventually. Like, who—where do we go from here? So, on first glance, it kinda scares me, to be honest.

3: Can I ask what you mean by a virtual primary care?

Moderator: So, a virtual—that’s—what we meant was, here, that it’s an artificial intelligence system. It’s not telehealth. You’re not necessarily seeing another individual on the other end. It is AI, which means that you will be interacting with a system that might ask you your symptoms and all of your demographics, and it’s gonna put it all into the computer, and it’s gonna come up with a response. That’s a very, very simple way of saying it, but another words, it’s not an individual.

3: Okay.

Moderator: Yeah.

3: That’s interesting. It really is. I would say, with that in mind, that my view is absolutely there’s room for that. Absolutely there is. How, where it could fit. It seems to me, like, you would have to be finetuned to the community perceptions of AI, and the limited applications of AI. I’m thinking Watson here - so, some things might work really well for Watson. Upper respiratory infection or something like—basic things where it’s just you have all this data, there’s a rhythm, there’s a trend. It cuts time; it saves money. I’m thinking of my people back home. If they can be sort of affirmatized to the idea of sort of like this library of human information ‘cause I’m sure if it’s an AI for health, it can also be used for other things as well. Some I thinking – How do we send that to Ngong Hills where I’m from? I would think maybe just introduce it, first of all, by sharing what it kinda does. You know, just a wealth of information, and show small ways it can be useful for basic things. Like, it would lead to the human element of—there would need to be—it would need to be sort of cloaked in the garb of accessible humanity for it to be something that folks who are not used to that idea as a normal experience for them to get comfortable with it. I don’t know if I’m explaining that well. Like, if you’re gonna drop it in the village somewhere, there have to be people in those instances to steer it to the people - there would have to be people from the village who are respected by people in the village or who can stand in for people who look like them for whom this virtual experience with this machine is sort of like a representative, who have to see this as a reflection of the people they love, know, and trust for it to work. But functionally, I think it’s a wonderful idea. But I think you would need to have very clear limited applications, at the get go, I think. Yeah, that’s my - that’s my thought about that. And his thoughts are lumped in there too.

Moderator: Yeah. So, I hear—I hear convenience, and possibility. I hear absolutely not, and what I was hearing from 1 and a little bit I think, 3, am I saying that right?

3: [name].

Moderator: What you were both saying is that there has to be a human element. Am I understanding that right? Maybe as a next step or how are you envisioning that?

3: I think, I think at the same time.

Moderator: At the same time.

3: Yeah.

Moderator: Okay.

3: And I’m thinking in Kenya, like, we don’t have electricity, but we have technologies that are being brought. So they’re like, forget putting lines in. Let’s get everyone LEDs with solar power, so you get power without the infrastructure. And this seems like a way of getting people who don’t have access to help their healthcare, without going through the bureaucracy of getting a doctor and a clinic—I’m sure, like, having some sort of semi-working clinic. You know, that’s how we see it. That’s how it’s use in that space. I don’t know if I would use it here [laughs]. I’d use the care as tool for education.

Moderator: Okay. Any other thoughts.

2: Yeah. I would feel comfortable with a tool of this nature especially given the convenience component of it with—but I’d have to know that it was—I’d have to know who designed it, and I’d have to know if there’s some sort of in-built accountability. Like, is there a way for me to contact someone if I—after the algorithm gives something, but yet in my mind I’m like, no. I think this might not be it. Like, is there a way for me to contact someone or would I just contact my regular physician? Like, I’d want to know what those steps would be to feel totally comfortable with the tool.

Moderator: Are you talking a little bit more about how it works? What’s behind it, and what to do with it?

2: Correct. That would be a critical component for me to get to comfort level where I’d feel comfortable with the tool. Yeah.

Moderator: So, what would it take to feel comfortable with a virtual -

2: Knowing who designed it and whether it was a physician or a group of physicians or a hospital or was it—is it—what dataset did they use. Like, I’d want to know more about that.

1: I’d wanna know it’s limitations. I’d wanna know what am I using this tool for? Like, do I present all cases to it ‘cause I mean I can’t speak for everybody, but I would assume most people go to the physician for two reasons. One is they know what they have and they wanna get a medicine, or they don’t know what they have and they’re kinda scared. I would not wanna take that option to a virtual PCP. Like, I would not trust an algorithm to decipher something that I don’t know what it is ‘cause, like, I’m havin’ a hard time seeing the difference between this being, like, Web MD, but you don’t have to search out your own answers. You can just tell it, and it’ll spit out an answer to you.

Moderator: Yeah.

1: I definitely—I think I would have to know the limitations, and I’d have to—I agree with 2 a little bit about if I’m given an answer that I don’t—that I wanna have some shared decision making with who do I—do I go back to the algorithm or is there somebody I can contact for that? That would be important to me to know that.

Moderator: Mmm hmm, please.

4: I think from my perspective, especially given our recent induction into epidemiology, I would really need to know the effectiveness of it too. So, if you have this artificial intelligence, you obviously have a training set of data that you’re presenting. You have to sort of feed it data for it to develop its own ability to discern patterns and things of that nature. I’d like to know how does it do, you know, when we test it on other datasets, large datasets, comparatively with perhaps the best primary care practices in the United States? Diagnose accuracy, things of that nature. So, if I know that it’s an effective tool, it’s been proven, it’s been vetted, that would make me feel more comfortable.

Moderator: So, that’s kind of similar to what you were saying.

2: Mmm hmm.

Moderator: Okay. All right.

5: I think I would need it to be able to respond in a way that I am acclimated to. So, if I understand that it’s going to be responding in a very artificial way, emotionless, I would just wanna be prepared for that response and expectation, but I wouldn’t want it to—I guess my concern is if it told me that I was about to die, that it did so coldly, and as a matter of fact. I think I would want it—for something as serious as that, I would want it to be cushioned, like, the way a human would. And the way that it says certain things, need to be different depending on the situation. So, I’m thinking about this movie that we see where a patient was told he was about to die, and this robot just coldly drops medication in front of him and says, “Take these until you perish.” And I wouldn’t want that of a virtual PCP, but at the same time, I’m not sure if it’s better to have a fake visualization of a face. Like, there was an AI simulation that was used to catch child predators over the internet, and it looked very life-like, and spoke very life-like, and it convinced predators to approach it, and they were able to find these people, and use that tool. I don’t know if I would want that either though. I think I would just need to know and be sold as a consumer the parameters that this will exist in. Whether it’s going to speak very artificially or be very realistic. I’m just not sure what I would want right now without seeing the technology.

Moderator: But that’s good because we’re gonna come to that, but just so I understand. When you say you mean that it sounds like a robot, or do you mean it sounds like Alexa? I mean is it a—what would make it more attractive for you?

5: I think that it had natural speech patterns, spoke like a person, and was able to at least strike a tone of emotion at the right times, but maybe not always. I don’t always need it to be chipper. I don’t always need to be stressful. I just need it at the times when it’s necessary that it will know that I am feeling a particularly devastated way or particularly that I want it to be able respond to that.

3: I would want it to be - I would want it to consistently in some way remind me that it’s not human. I don’t want to make the mistake that this is human. I think— that sounded a little – I’d just want – like, I know Alexa isn’t. Like, I know that’s some extension, but it’s not… And I was thinking about that as 5 was talking, and I was thinking to myself. Actually, you know what, I don’t want it to have the answers. I want it to be a reasonable and, well, take reasonable, and I don’t have the words for it ‘cause I lose my brain when I’m on the spot… But I want it to be able to say, I’m getting this from the BMJ. But for a second opinion, you can go somewhere else, or I want you to, you know, or this is what – and, you know, it gives me a way that I can get a second opinion, almost [inaudible]. Ah, there was another thing that I thought was important to me…

Moderator: So, are you saying that, like, kind of what 2 and 4 were getting at, that you wanna know the sources? You would wanna be able to ask?

3: I’d want it, unfortunately, to be vulnerable.

Moderator: You want it to be vulnerable.

3: Yeah, I know that’s really—it’s humanizing it, but I want it to be able to say, I don’t know or I’m uncertain about this, or I think this is what’s going on, but I think in some way it’s important for you to get a second opinion from someone unbiased in their opinion, this is where I’m getting my sources from, and in a way that reminds me it’s a tool.

Moderator: So, it’s not supposed to pretend that is a human provider.

3: I think so.

Moderator: You want it to be clear that it’s a machine.

3: Yeah.

Moderator: Okay.

3: And my thinking there is I need some algorithm to get differentials, so I use the BMJ as a starting. I use all these sources to get what I need. I’m not on social media, but a lot of people trust their lives to algorithms: Tinder, Facebook, all that stuff. So, I don’t think it’s a hard sell on that front, but it has to be a limited sell. I also kinda wanna know—I don’t want it to have personal information. I don’t want it to have HIPPA stuff. I just want it to be an information giver. As I was thinking, I was thinking extrapolating, going, well, you know if it does have that sort of HIPPA information, then I guess there’s gonna be some federal oversight on this machine. And who knows what they want to check and what they don’t want. And I’m not trying to be like, spacey here. But—

Moderator: No. No. It’s good that you’re thinking of all of the—these are all aspects that are—

3: So, by the time it shows up it may not be what it was intended to be, so why not just limit it from the get-go.

Moderator: Okay.

3: And just say, like, start off with just being an information tool.

Moderator: So, what advantages do you see with a virtual PCP? Are there any? I mean you mentioned it can be convenient.

1: I see this as like the next logical step in telehealth, so I’m thinking, like, this sounds a lot like I can pick up my I-pad and talk to a physician on my I-pad, right. Like, so I’m imaging, like, this is just the next logical step in that direction. I think the same rules apply in terms of advantages with that support of telehealth, with the virtual PCP, so convenience. Probably cost, reduced manpower in terms of, like, the physician staffing and things like that. Like, okay, like we can jettison this portion of the patient population off because they’ve gotten their needs taken care of already. So, I think those are all pretty good benefits in terms of a corporate side of things.

Moderator: Mmm hmm.

2: I actually also see, in addition to that, I see benefits in communication. Sometimes when I see my doctor given the limited time and the emotions that I feel at the given moment, I’m not able to communicate everything that I intended to communicate prior to going in. And also, I think that’s one of the challenges in finding a good PCP is finding someone who you know can communicate with you, and will pick up on all the cues, and to some extent, like, you have to be okay with not placing such a high expectation on someone else. But the one thing that tech and tools of this sort circumvent is that noise that comes in human interactions sometimes. So, it’s easier to type out everything that I’m concerned about versus, like, verbalizing all of it, especially if it’s emotionally charged or embarrassing or whatever it is.

Moderator: So, you are—are you envisioning that it’s non-verbal? Would you prefer that interaction to be non-verbal? You know, that you put in your question or you put in your—

2: I’d like that option.

Moderator: You like that option. Okay.

2: I would like it, and I also see that being a benefit for—I have a family member who has an intellectual disability, and so that would be—and see communicates way better texting than she does in person. And so, I’m also envisioning that it would be much—it would be useful for her in the future.

Moderator: So, that’s exactly what I was gonna ask whether there are any situations in which you could see that this could be actually an advantage over an ordinary face-to-face. Okay. So, that’s a great example.

2: Yeah. And there’s also certain assumptions that people make when I go into a room, like they assume that I’m quiet, or like, there are certain, unfortunately, certain stigma or certain assumptions people make about people based on how they look. And that’s kind of why you can pass that. You don’t have to deal with that when you’re dealing with a tool.

Moderator: Mmm.

2: I mean it has limitations, like, we’ve been discussing, but those are some advantages that I think can be leveraged.

Moderator: That’s definitely – yeah?

4: I just wanted to add from a research perspective, it would be revolutionary. Data abstraction from patient charts is now done manually, and this could automatize that. And also, in addition to that, epidemiologically again, certainly better, we could predict real-time health outbreaks that are occurring. So, if let’s say there’s a case of E.coli contamination in lettuce, and everyone that went to Chipotle, you know, got contaminated. Yeah. I could query everyone that calls in, did you go to Chipotle? And all of a sudden, we have epidemiologic data that suggests, oh, in this region the outbreak is concentrated. It was this batch of lettuce that was contaminated. We can trace it back, and so I see a place for that. And also, in terms of just research in general, collecting basic patient data, demographics... I mean if you think about all the different studies that are conducted and all of the mounds of data that you have to collect, just on patient demographics alone, this would really speed up that process. And you could have a centralized pool, and all researchers could have access to that data. And so, all of a sudden, when you’re doing a cohort study, your N goes from 100 to 100,000.

Moderator: Mmm.

4: So.

Moderator: Definite advantages.

2: And actually, on that note here, as someone could potentially use that tool, if I knew that that algorithm did that, it would make me want to use it more, right? ‘Cause, oh, my, gosh , is there an outbreak going on in my neighborhood that I’m not aware of. And then I will, like, type in my question, or I’ll—so that would be interesting.

Moderator: So, do you foresee that as being a possibility? So, you’re contacting the PCP with a question about your community rather than about you, yourself.

2: Mmm hmm. Right. It would be a way to contextualize what I’m experiencing. Like, if there is potentially—if I know that this tool or this virtual PCP has an algorithm for that, and if I have a certain symptom, that might prompt me to use it more frequently.

Moderator: Mmm.

3: I wasn’t thinking that, but thank you for—I was somewhere else, but then I was thinking, oh, God, yeah. So, if something happens, like, what’s going on in Mozambique right now, with the flooding. In countries that are, um - it could potentially figure out, based on what’s going on with the information, what it specific needs are, like in the case of supplies, for that area, and it could be a mobilizing tool. Instead of, oh, I wonder what’s going on, if you can get enough information about, like, so then you could maybe algorithmize so we need this many IV bags, and so much of this antibiotic or that or the other and maybe predict and have folks who are heading over there learn how to get there. So, thinking in wider angles, it makes me nervous too, could it be used for harm? Who’s in charge of it. It’d almost have to be, like, a UN kind of thing, which -

1: I guess if I could just piggyback off it, I’d certainly like—we certainly live in era of big data, but, like, that I think I would not be okay with sharing my information. Like, not even—I don’t care if it’s depersonalized. I don’t care if a number’s attached with it without my name. I wouldn’t wanna share my information with anybody. I mean my health information is my health information, and right now the way the system is built, I sign a contract that has a privacy statement that I can read, and you could surely put that into, like, an app or something like that. But, like, the big data aspect of things, if that would—I would intentionally not use a tool like that if I knew that that data was being used like that.

Moderator: Could be used. Okay.

1: Yeah. For sure.

Moderator: Do you see any advantage? ‘Cause you didn’t like this idea from the get-go.

1: I’m playing like the devil’s advocate here, I think.

Moderator: I’m just trying to see are there any other—are there any positives?

1: Totally! I mean I think, like, I think convenience, I’m sure the cost would be really low.

Moderator: You mentioned cost, yeah.

1: But I actually like 2’s thoughts, too, but I totally agree that 15 minutes is not nuanced enough to hear somebody’s story. And so, I think this gives people a different avenue, and it may be a different form of communication to share that story. So, I really do like that aspect of things.

Moderator: All right.

5: Also, think about it in the context of mental health, so one of my biggest—well, one of my understandings around deficits in medicine is that there’s a lot of mental health need, and there’s just not enough providers that can give it. And I feel, strange to say this, but maybe we need more digital AI therapists to, like, guide a conversation, listen to what someone’s saying, respond to them intelligently, but that might be really complicated as an algorithm to, like, listen to conversational things and pick up cues, pick up—maybe this person isn’t mentioning everything. It’s a very complex subject, but I just wonder if the time spent for the patient to hear themselves saying these things, and to just say them out loud and verbalize them, is just as important as having someone hear them, and the application’s there would save person-time, so they can free up other professionals to continue doing other things. It could increase accessibility, so, like, we were saying in a globalized world, more people have access to more a more standardized level of care. But it also lets us really free up and think about what are the possibilities outside of what we’re already doing because I feel even just outside of medicine, we’re kind of limited to just slightly, incrementally building and maintaining what we have instead of looking and seeing what else can we do, and what can we tackle if someone else can do the base level stuff? And I’m not saying that being a physician is a base-level thing at all ‘cause it’s not—it’s a lot of work, but it consumes a lot of our time to maintain and do, and we’re just not able to do all the things that we wish we could as a civilization, as a race, as a species. So, I think there’s potential for us to then step forward further if have this base that could help us keep building up.

Moderator: So, explain a little bit more what you—are you saying that a person with mental health issues could use this as a first step or as a substitute for?

5: I think it depends on the case.

Moderator: Okay.

5: There’s definitely degrees of severity where someone with a very acute potential to harm, might not be, I think, the best patient for this case. But if someone had anxiety where they wanted to talk out certain problems they had, and there’s a list of questions that would help them prompt and talk about their thought process or their motivations behind things, to have them be walked through a series of questions would probably be just as helpful if not more helpful than—I’m sorry to stereotype it—like a therapist sitting there doodling while asking them questions.

Moderator: Hmm. Okay.

3: Can I piggyback on that? I was thinking I worked in the psycho-school for a while, and a couple of things frustrate me. One is a flexible algorithm for determining who is suicidal and who isn’t. And that got me also thinking about cases, like, some counselors that are really hard to get to, and I got to thinking—and the other thing that kinda gets me, too, is this peoples’ rights to die. I don’t know why that’s important to me, but I almost feel it’s something that I think certain folks who are towards the end and staying is not an option that’s feasible, but there has to be some room for quality and conversation about that, which then becomes how do—where do you place the market value in that - the direction, you know, especially when they’re suicidal. The person has a—he’s mentally present enough, and is in enough pain, and he’s about to die, so that decision is okay. And I was thinking if you could feed the program as many options and versions of, you know, mental stress as possible, then maybe I could go there and just kind of say I’m a convoluted, half-broken story, and it could potentially popup the salient themes that are in there, and that could actually make clear for a more robust definition for this person who really is suicidal, or this person really is just having a hard time in life right now, and they’re using this idea as a way of sort of asking for help, it seems. So, for the nuanced things that probably require larger crew of information about this one particular situation, is a program that could get all those little bits and pieces in order to create a picture, and then I could just walk in and just say what I have to say, and it could, within the context of mental health, make a fair judgement and assessment that can be checked by someone else. Say, I think this guy seems to be suicidal, and he might need to or want to talk about this. So, as a tool, as a tool, I don’t know if that made sense.

Moderator: But you see that as a clear advantage to having that algorithm in place?

3: Yeah.

Moderator: Rather than relying on one individual’s interpretation. Am I understanding you?

3: I think they would have to be together, but I think—

Moderator: Yeah. Okay.

3: Yeah. I think definitely as a primary step, it could easily save—based on these, these, these, or these things that are within my system, I think you need to look in this direction, like, with this human is having a hard time expressing how they feel, then then you can use that ‘cause a lot of what I saw was just gut feeling that was just not valid or—so, that’s my... But that’s my thinking about the nuanced things. This is an assisting tool.

Moderator: So, now we’ve already touched on this, but what are some of the drawbacks and situations where you absolutely wouldn’t be willing? And I know we’ve already heard some of those.

2: In regards to mental health, I actually would prefer not to use AI because I would feel even more isolated than I think I would if I was in a state where I was mostly dealing with depression or anxiety. It’s like, I’m still alone [laughs], and that’s a big part of core mental health. At least when it plays out in my life. Even though I know that someone else is maybe not picking up on the cues, the act of being in a room with someone who’s is more—who is trained, that in and of itself is therapeutic and helpful. So, in those I would prefer not to use the AI.

Moderator: So, that differs very much from what 5 was saying.

2: Mmm hmm.

Moderator: So, you can see both sides, it sounds like. That it can be cold and impersonal and not helpful, but on the other hand it can maybe open the situation to speak freely because you’re not being judged, or you don’t have to worry about the personal connection. Any other drawbacks, if we think about other types of illness other than mental health?

4: I think one of the biggest drawbacks, and this is something that is in medicine in general is patient dishonesty. So, unfortunately, physicians do have to deal with a small subset of the population that comes to the doctor’s office or the emergency room and comes up with a presentation, essentially, “Oh, I’m sick”, or “This hurts or that hurts,” in an attempt to procure pain medications or other things, or some people may have psychosomatic disorders or things of that nature, and so I think someone brought up litigation, which is a really good point because what if you have someone who fakes symptoms of pain, this dispenses an opioid, and then who’s at fault? Definitely drawbacks.

Moderator: Interesting. Very interesting. Other drawbacks you can think of?

1: I guess, like, I’d say—I agree with U about the mental health thing. I can’t think, actually, of, like, a worse use of that tool. The mental health one of the most important things is being in the presence of somebody else, and knowing you’re not alone, and I think a virtual PCP would further emphasize the fact that you are alone. But the other thing I see is just the complete lack of a physical exam. I wouldn’t be examined. My heart wouldn’t be listened to, my lungs wouldn’t be listened to, and certainly we could get into the merits of the physical exam. How much does it actually pick up? How much does it? But the complete lack of touch, the completely lack of a physical exam would bother me.

4: I think another point that was mentioned earlier is data security, so whenever you have the collection of protected information whether it be health, passwords, bank statements, otherwise, you have to have a method to secure it. And we know that identify theft is extremely common, and so personal health identity theft I feel like is going to be one of the emerging issues in the future. And so, having a robust security network that protects the patient information that was collected is gonna be crucial for the success of any sort of artificial intelligence or data collection program because as someone said, people not only should have the right to opt out of data collection, but they should also have the right to know that their privacy’s being respected.

Moderator: So, would you envision that an AI system would be less secure than your primary care physician’s EHR system, or not necessarily? You’re just sort of—

4: I’m just positing that as a potential drawback.

Moderator: Potential. Right. Okay.

4: When I go to the physician, I have a conversation face-to-face. If I decide to share something with them that doesn’t make it into the electronic health record, that remains confidential. If I am speaking with an artificial intelligence program, anything that I say gets recorded. If all of that is collected, and there’s no way to sort sift through and parse out relevant details, then all of that is collected, and potentially is at risk for being accessed by someone who I don’t want accessing it.

Moderator: Any other drawbacks that you can think of?

2: The drawback I see is if the system’s closed, if it’s not constantly developing, that would make me feel very nervous.

Moderator: If it’s not dynamic, you mean.

2: If not, mmm hmm.

Moderator: Yeah.

2: Because one advantage of having a human—a real PCP let’s say, or a human PCP—is that person is somewhat trained to know where their knowledge is limited, and to use their imagination. Like, there is—when it comes to development of medical knowledge, there is always that component of the unknown and people looking for other answers that people haven’t thought of. And if I was dealing with a system that is a closed system, and in my mind when I go to sleep at night, I’m like, but wait. What if it’s this random thing, and that didn’t even like account for it. Like, I would not be able to sleep at night. And I would prefer a doctor who was like, “I actually don’t know, but let me look into that,” and then they talk to someone else, and they talk to someone else, and talk to someone else. I’d prefer that, and there was something else in addition to that, another drawback that I saw. I will mention it if it comes back.

Moderator: Okay.

2: But that’s one drawback I see. If it’s a closed system, a closed algorithm, no way to be dynamic, I get very nervous.

Moderator: Would you feel better if the system said, I don’t know.

2: Ah, yes.

Moderator: Okay. So, that would not be a drawback?

2: Uh-uh.

Moderator: Okay.

1: I can say, if I may too, another drawback I see is—so, it’s AI, so it’s gonna be very, very complex computer technology, and so I worry we’re gonna lose the clinical aspect of things because it’s gonna be developed by technology companies. I’m imagining in my mind this is, like, Steve Jobs coming out in 2030, Apple Summit saying, “Guess what. We have, like, Siri for the body.” And like, I know that that is gonna require a lot of computer programmers, and not a lotta clinicians. And so, I worry that we’re gonna lose the clinical aspect of things because by its nature it’s gonna be a technology and not clinical piece.

2: Going back to that one—that other thing I wanted to mention was one concern I have is that if this tool is able to pick up a lotta data, and the tape which picks up data is faster than the pace we would—a clinician or someone who’s experienced—could review it, I would get very nervous. ‘Cause then it’s almost like I’m now trusting a system that doesn’t have a brain, most humans have a brain, like that would get me very nervous. I’d want to know that this data or this conflicting review that’s being vetted by real people and not developing its only life. Otherwise, how would you check on that? And I’d feel very nervous about that.

Moderator: So, I think you’ve come back to actually your original—your very first comment was that you wanted to know what’s behind this? Who’s behind it? Who’s developing it? It’s a little bit tied to that. Am I understanding your right? Yeah.

2: Yeah.

Moderator: And who’s keeping it going? Okay. So, kind of a verification or a validation process, plus what did you call it? Kind of a keeping it active, right, rather than static.

2: Yeah. Instead of a closed system.

Moderator: Okay. Not a closed system. Okay. Other?

5: If I can interject, I would also wanna make sure that the AI itself is at least to a level of standard of care that is equal to a human provider. I wouldn’t want it to become a striation of you can’t afford to see a real person, so you’re gonna be here with this computer. And you can afford to see a person, so you’re gonna be over here. Like, in terms of your outcomes of care, I don’t want there to be a stratification of, oh, the poorest people see the computer, the richest people see the person.

Moderator: Mmm.

5: I would want it to be that anyone can get the same or just as good level of primary care from this machine as they would a person. I don’t want it to be a subclass thing. Like—

Moderator: Like, all the Medicaid patients get the virtual PCP, kinda thing.

5: Yeah. I wouldn’t want that to happen. I’d want it to be broadly applied.

Moderator: So, should—it actually, a little bit, brings us to the next question, which was how you envision this? How would you envision actually using it, and that—you raise a really interesting point. So, should a virtual PCP be available to everyone, or just to those who choose to use it? Should it be part of the system? Should it be, you know, a cog in the wheel? Is it a step in care?

3: Who’s paying for it?

2: Who’s paying for it?

1: Yeah.

Moderator: Okay. Well, that’s part of how you, you know, how would you envision this? Is it something that you pay for out-of-pocket? It’s wide open. Whatever you think.

3: So, you’d need a cloud service for it.

Moderator: Okay.

3: So, it means it needs Amazon. They have the biggest cloud service out there. And that would be my, where I don’t know that much about it – about the big names, about the money. So, in the end, I don’t know. When a machine is present, who owns the machine?

Moderator: Mmm hmm.

3: So, I’m envisioning care, right.

Moderator: Yes. That’s good.

3: So, I don’t—I’m still not encouraged to look beyond the idea of it as a tool. If it’s already gonna come with a lot of like, with the equipment baggage, with the law baggage, probably, and I hope at least with some kind of oversight, so like a national law. I think as a tool, then it should be made as open source and accessible to everybody. Not necessarily as a stand-in for a physician. I just see it as a way of having 10,000 brains about this one problem as opposed to just one. So, that’s why I don’t want personal information in there. I want ideas and I want a lot of forward thinking there… When I visit, these are the stats about this -

Moderator: You’re thinking of it more of kind of a global looking at population health rather than for you personally. You wouldn’t wanna put your personal data in there.

3: I think by the time we get there, get through all the red tape, the bureaucratic stuff that we don’t wanna get into. We’ll see what comes after original 1.0. What does version 1.2 look like? If we’re moving away from looking up something online to envisioning a virtual program or that can be on telemeds to a virtual program, what comes after that? And will it align with the feelings of the people? I think there’s a possibility 5 or 10 years down the road that the overuse of the phones, people will be like, let’s move away from that. Let’s move back to being just humans again. Will it be viable then? That sort of thing. Which it will actually have to have a soul —people who are designing, would have to have a forward-thinking soul that adjust this virtual experience to fit their zeitgeist, is that the right—

2: Zeitgeist.

3: Yeah. Keep up with the pop culture and that stuff. It has to be able to adapt to the experiences of the people at that time when they use it, you know. So, I don’t—my name is not going anywhere . Keep my address—only my address.

Moderator: What if there were a way to monitor your vitals through this machine either with a chip or a blood sample or looking in with, you know, your eyes or a finger—how do you envision that this could possibly function?

1: I envision, like, uhm, okay, like I’ve used Blue Cross Blue Shield, and I know that with my insurance company, I get one free, not really free, but it’s free kinda, one free, like, visit to a physician, right. And so, I kind of envision this as, like, I’m sure Blue Cross would love it. They could send me a little machine or, like, the app on the phone, if it’s—I don’t know how sophisticated we’re getting. And then as part of my gold package, I can use this machine or this app five times this year if I need to. So, I envision that, and I agree with the tool use. I think it certainly would be more of a tool for me, but if I was really curious ‘cause my mom’s diabetic, and I’m a little bit worried about her blood sugars, I could just do a quick scan. Use it, like, one out of the five times for the year, I could see a benefit to that. Yup.

Moderator: Okay.

5: I don’t think we’re that far away from having continuous vital monitoring. I mean with the Apple watch stamp being studied.

Group: Yeah.

5: —the potential of the watch and people seem to be very willing to do that kind of real-time, 24-7 monitoring as long as it provides them some kind of benefit. So, in this case, it’s notification. So, if you could put in a form that is useful to them personally while also in the background monitoring those things, I think people will be all for plugging in.

Moderator: So, you’re talking about monitoring health, right?

5: Yep.

Moderator: What if you’re monitoring disease? Kind of like what you were getting at. What if you’re diabetic? What if you suffer from anxiety? What if you have a certain kind of cancer? Any other chronic disease—heart failure—are there benefits—do you envision ways of using artificial/virtual for that?

4: I think it could be revolutionary, like I said before. You could have someone who’s a diabetic, and you could feed the artificial intelligence data about your last blood glucoses and your A1Cs without leaving your home. Just a simple, you know, if you have a home machine or something like that from Blue Cross, it’s funded by them, and it collects all that data, and then it gives you risk estimates for development of chronic kidney disease, which is the most common complication of diabetics—long-term diabetics. So, it could easily tell you, your blood sugar’s lookin’ quite good these past few months. Your risk of kidney disease is X. If you were to eat healthier, your risk would be even lower. And so, it could prompt people to take additional measures for their health or it could warm them. I’ve noticed that your blood sugars at night have been getting higher and higher, and you should curtail your snacking habits, in that case. You know, so there are different trends and sort of patterns that it could analyze and preempt to prevent complications of disease, in that sense. And because you could—I envision it as something that you could go and use every day, as many times as you want. I wouldn’t wanna put a limit on it because the more data you feed it, the better it gets at predicting, and then the better it gets at predicting, the more accurate it is. And so, in my opinion, it would need to be used more and more for it to actually be worthwhile.

5: But if we keep doing that—if we keep along that route—I’m just projecting here—but what would it—would it eventually lead to the robot saying, definitely do this, don’t do that? Do this, don’t do that. Here’s how you should structure your day. Here’s what you’re gonna eat. Here’s what you’re gonna do at this time. Because these are optimal for your health. Is it gonna project and tell us what to do, and how to live our lives? So, with the ultimate goal, which is to live long. Is that the goal?

4: In my idea, I guess, or the way I envision it, is it would give you recommendations and suggestions. But at the end of the day, you have to imagine this is an artificial intelligence program. It cannot force you to do anything. It can tell you a 1,000 times, don’t eat that. Don’t eat that. Don’t eat that. Ultimately, we are human. We do what we wish. We have free will, so I think—and therefore the limitation of it is it can suggest all these great things to you, and tell you what you should be doing, but that doesn’t mean the patient will be compliant. So, another drawback, again, just patient compliance. But if it were to do it politely and nicely enough, you know, when your mom wakes you up out of bed by gently shaking you versus pouring water on you and yelling at you, you’re more likely to listen, and kind of have a positive attitude. So, I think it’s highly dependent on that. I wouldn’t think of it as issuing commands. Must eat low-carb bagel.

5: I think if a computer program suggest to me that based on this one billion population set of data that showed that this outcome was or this action was associated with this outcome for this percent chance, it would almost be saying to me, like, you should definitely do this. Like, of the billion people that did this 900 million of them benefitted. That’s a big suggestion.

1: If we go down that road too, even if the tool—you may not want or need or not have to follow the tool. I absolutely, 100%, see this being used with health insurance, if it still remains privatized with the government and with employers, saying, like, if you wanna buy our life insurance, or you wanna buy our health insurance, you need to follow the guidelines of this virtual PCP every single day. You need to checkin, and you need to be compliant. And if you don’t, then you’re out of health insurance. You know what I mean? Like, I totally—even if the—you don’t wanna follow the robot, well it’s too bad ‘cause, like, Uncle 1’s tellin’ ya you need to or you’re out of a job. You know, I can totally see that being used.

Moderator: So, where you would actually—are you saying that you would actually have to do what it says and check-in every day, and it isn’t just a question and answering, yes or no, but you’d have to give—

1: I wonder. I mean I think right now if we—even today there are employers and, like, they have good intentions, and then maybe the benefits we know are pretty big, but like, they’re not gonna cover you with the company health insurance if you smoke. And they’re gonna check your blood to make sure you don’t smoke.

Moderator: Right.

1: And so, what’s to prevent them from using this tool even more invasively in our lives?

Moderator: So, we’re gonna wrap things up. Could this be a reality? Do you foresee it as being a reality or is it too out there?

2: I want actually to build off of what 5 and 4 were talking about in terms of this algorithm providing suggestions. I see this being very helpful for individuals like myself who often find that after you go to a doctor’s visit, like, there are caregiver roles that you have to play for family members, and if that work can be done by the tool, it could actually be very helpful [laughs]. Instead of me reminding a family member to eat healthy or to remember to take their medications, this tool would provide those nudges. That would be very helpful.

Moderator: Would that happen without input? It would—

2: There would have to be some—

Moderator: It’d be programmed to? Okay.

2: So, we could deal—it could alleviate some of the caregiving burden that unpaid caregivers have to already put in because of how strapped physicians already are in terms of what they can do. So, I wanted to make sure I mentioned that.

Moderator: Mmm hmm.

2: But I definitely see this as a reality, in some shape or form. There are drawbacks, but I think the advantages can be leveraged to some degree to offer some benefit.

3: At the county jail where I’m from, you can’t have visitors in person anymore, and the prisoner who is in front of the screen, when you stand in front of a screen, and that’s how we say when we, you know, that we’d be visiting.

Moderator: Wow.

M: So, thinking that this tool can be used in a jail. I would think that would a place where we need humans more. I can say it could be used as a possible tool to try to dehumanize people. At the same time, being used as a possible tool to help caregivers who don’t have access. I can see it being used there in ways that don’t help. I think if it does go—have federal oversight or government oversight, who’s to say they can’t do that same thing to patients. That’s another one of those painful thing I saw when I finally saw the person I was visiting. And 2 years ago, I could see him face-to-face.

2: And I could actually see that exacerbating disparities that we know already exist, so that’s also one risk that we run with tools like this.

1: I do agree it’s inevitable though. I think there’s too much money to be made and too much money to be saved with this tool for it not to eventually come to fruition. I think there’s so—like, there’s huge potential for corporate interest in that. So, I feel like it will totally come to fruition.

Moderator: So, will physicians be out of a job?

2: Nope. I don’t think so.

M: [Laughing].

M: No. I do think that—

2: You think so?

M: Or not out of a job, but I think that physicians are increasingly going to play different roles in peoples’ lives. I think they’re transitioning from the one-on-one caregiver to the patient care management role, and this would further that. I think we’re already seeing that today, but I think this would be more of, like, less often going to see a physician face-to-face. More often the physician is managing data, and kind of organizing data, and I don’t know. I kinda think—I think it would change physician roles a lot.

2: Hmm.

5: Can you remind me the initial prompt for this question ‘cause I think I got lost?

Moderator: The initial prompt was could this be a reality?

5: I think in our lifetime AI probably be more of a tool, like a sidekick assistant rather than an actual independently operating—[inaudible] entity. I just don’t know how quickly technology will develop, but I doubt that our generation will be willing to conform to that kind of change and experience. I know that we’re not too far away because so many of us interact digitally already, and when we’re sitting physically with someone, we’re already trying to interface with someone not physically here on our phones, or just look up something. So, I don’t see it being too far outside of the realm of possibility, but I think—and I’m gonna put my potential provider hat on—I don’t think I would want someone else—something else to do my job for me. I think I would like to consult with it. Maybe use it as, like, a reference and figure out, okay, that’s something I hadn’t thought about before. Thank you for providing that, but I don’t think I would want it to do everything. There are some things I might like it to do, like, hey, I have these other 10 patients. Tell me who’s most likely to have a problem, and then I’ll make sure to focus when I talk to them. Whereas these other ones, if you’re telling me that nothing’s—nothing’s abnormal, I’ll just go have a talk with them. Tell them their physical looks great, and then also check it over myself, obviously.

Moderator: So, that’s an efficiency tool.

5: Yes.

Moderator: Yeah. Like you would also use it for differential diagnosis.

5: I think so because I can’t say that I’m gonna—I can’t say with great confidence I’m gonna remember every single condition or every test and every variable that things can be measured and know what it means. Hopefully, at one point, step one will—all that knowledge will be inside here, but not today.

Moderator: Okay. Any other final comments before we conclude? Any other thoughts?

5: Can I say that a lot of my hopes for the future revolve around kind of like the Star Trek aspect where it’s a very utopian society. It’s basically socialist. There’s no money. People do what they want because they want to do it, and they’re interested in pursuing knowledge, all these things, and at some level there is an emergency medical hologram that can fulfill these functions, but it is not the primary healthcare. But someone could use if they wanted, or if there was a need where the physician’s out, and the physician’s incapacitated. But it has the same level of emergency training to address acute issues, so.

Moderator: So, you do see a place for it, it sounds like. You envision that it could fulfill some role.

5: In the future.

Moderator: Yeah.

5: I don’t think in our lifetime, but in the future.

Moderator: Okay. And when you say not in your lifetime because you think there’s so much more technology that’s needed to make it work?

5: I think that there will need to be more of a—I don’t think the robotics are there.

Moderator: Okay.

5: I don’t think the physical processes that a provider does can be replicated by a machine right now. I think the different procedures and aspects of medicine that as human providers will be able to provide can be minute—I mean singularly done well be a single machine, but not by one machine doing all these different things. I guess, like, you can make a machine that’s really good at one particular function, like, maybe it’s chest compressions, right. Whereas, you can’t take that same compression machine and use it to then, like, a percuss different parts of the body. Like, our hands are very versatile instruments, and you can make the perfect machine to do one thing, but you can’t make one machine that’s perfect to do everything. I’m not saying that humans are perfect at doing everything, but we can do what we can with our bodies. I don’t think the machines will be able to do all those flexibility roles [6.31].

Moderator: Okay. Anything else?

1: I guess I don’t wanna go overtime. The only last thing I was kind of thinking of was I worry about the role of technology in our lives and how limited experience we have of it so far, so I mean, like, our modern world as we conceive it today has been the product of the internet, which is not even 20 years old now, at this point. And so I do think we’re gonna reach a point where we kinda, like, outgrow ourselves, and we’ll need to find our own place, and what it means to be human, and how to interact with this technology at some point. And I think somebody mentioned it before, but I worry about our generation in particular, but also generations to come here of how socially isolated we are, how we struggle with human interaction already because there’s so much screen time and so much technology time. And I don’t think we figured out technology’s roles in our lives at all. I don’t think we know, like, the role that we’re to play in that. And so, I don’t know if I’m just perpetual humanist, but I wonder if we might reach the conclusion that cell phones were, like, the worst possible thing we could have done for humanity. We need to backtrack or where we’re gonna go from here ‘cause we, frankly, haven’t figured it out yet. So, I think that that’s still to be determined.

4: If I could add something, the show “Adam Ruins Everything,” great show. It’s on Netflix. And he goes and debunks popular myths and explains historical context. And I recall one time he talked about jaywalking and car dealerships. And it was just so interesting because if you think about it, when someone created the car in Germany, it was a German person—the first person who created a car. Everyone must have thought this is madness. We have horses. Why would we need a motorized version of this? And yet, try to live your life without a car, so I think there’s a tendency for us to take these new things and think, how is this possibly gonna fit into our lives. It’s impossible. But we haven’t experienced it fully yet, and I think with technology we haven’t experienced it fully either. So, I think we should leave ourselves optimistic as to how it will change in our lives, but I really appreciate the points you brought up about the concerns and the drawbacks because nothing comes without negatives.

3: This guy I listen to sometimes talks about there was a time where we lived in a world of tools where the hammer was made for the person who wielded the hammer, and so the hammer was a tool, but also an organ, an extension of the organ of the hand. And then—and I’m sure all those people who are hammering, builders are ultimately not needed as much, and so some places in society it was debunk, like, maybe that was like 10 years ago too. But he speaks about how now we’re moving from that into an age of systems where we don’t know who we are because we are part of a system. And in a system, there is no I in paradoxical worlds; there is no we, and so part of the frustration he suggests that you hear that are people having with technology is because it at once is pretending to be your voice, but reminding you in the speaking that you don’t exist within—you don’t exist where all this internet. You know, like, we’re all the CHI. There’s no—some people are now trying to pull away from that and trying to say maybe I don’t need to respond to this email right now. Maybe I can pause and wait a second, and then maybe respond to it at the end of the day, or maybe I don’t need to be having my phone with me all the time. Almost to reclaim their place within a system that just will not stop, and a virtual sort of thing like this I think it can easily sort of obliterate the humanity of what we are doing ‘cause we are turning our bodies into tools that—one second—are heightened awarenesses of physical health, and sometimes. You know, the mental illness as well, and that’s a powerful task. It’s a big thing to do, but I also lose myself, and I become the public servant that is the physician, right. I don’t want a tool to make me less—I don’t want a special thing to make me less human. I almost feel like by doing what we’re doing we are becoming even more human than we used to be, and that’s why I would prefer for the virtual thing to be made a tool, so it doesn’t stand in the way of the space that I make between myself, and for instance, those systems that wanna suck me in, so that personally I don’t have the – Facebook, all that stuff. I have a flip phone that I would throw it away if I could, so I could have no phone, but I find this useful as like a storehouse of information, as someone has pointed out, that virtual use, but I think it has to be held gently, so it doesn’t exceed—seal the fate of the loss of our hearts or humanity. So, that’s what I feel.

Moderator: All right. It is exactly 7, so I think we will end there. Thank you so much.

2: Thank you.
