# Supplementary material for: Perceptions of virtual primary care physicians: A focus group study of medical and data science graduate students
Source: PLoS One. 2020 Dec 17;15(12):e0243641. doi: 10.1371/journal.pone.0243641 (PMC7745971; doi:10.1371/journal.pone.0243641)
Supplement: S1 File — (ZIP) [file pone.0243641.s002.zip › Anonymized Transcripts/vPCP Focus Group 2 Anonymized.docx]

vPCP Focus Group 2

April 16, 2019

Present:

Moderator

Two note-takers

Three 4th year med students

Moderator: This first question is really just a broad question of what is your view when—think, now describe this virtual PCP? It’s basically—it’s a machine; it’s not a human. What would your view be of using a physician in that way?

1: So, I guess it like it follows the—what seems to be the next trend in a lot of industry, right, is that more automation, more simplified things, trying to take some shortcuts as to, like, I guess you initially think of, like, being able to use it for, like, do I have strep throat? Do I need antibiotics? Do I have the flu? Do I need to go to the doctor? Like, do I need to pursue further care or is this just something that’s not very, like, important or severe or whatever it is? And then kind of thinking as a patient, I guess the initial concern that you would have is exactly that. That it’s a shortcut, and then you get concerned that, like, is this taking all the important criteria into account? Is this shortcutting, is this algorithm written right, right? Like, could this be missing something important that a real person would be catching? So, kind of having a little pause about using it is kind of my initial thoughts.

Moderator: All right.

2: So, from my understanding it would solely a virtual PCP. There would be no doctor associated with it. So, I guess as a patient, the closest thing I could associate to this is when you call up certain companies, and then they have a virtual a person or AI answer the thing. And most people’s instinct is to click zero and pound to try and get an operator ‘cause it’s much quicker. And then you say your date of birth, and then they’ll get it wrong, and then you try to say it again. So, in that regard, I don’t think it’s coming anytime too soon. Even on those phone calls, it ends up being a customer, like a—it’s a rep link, extension. So, they still have the automation to kind of get the route, but then you still have to talk to a rep at the end of the day.

Moderator: Eventually.

2: So, I don’t think that we’re even close to the point where you’d solely just see a PCP as a virtual. And the other thing is that as a patient, I don’t know, well, I guess I’d be looking from a clinician point-of-view, one second. So, I was gonna say, they would have less errors. It would be more likely that a machine would have less errors if you program everything correctly, but you’ve still gotta put the data in. And so, I feel like, it’s a lot of subjective data, so still the patient—a lotta times when you’re in—like, the doctor needs to cue you on what to say, and so I feel like it would be hard for it to have a robot cue you in on what else you need to say or how to describe certain things. It’s not—I don’t feel like a lot of medical things are just I have x, y, z. It’s a lot more subjective than that. I have had this happen for 5 years, and this, and I’ve had this stressor. So, I don’t think it’s just simple checkboxes. Like, med school we learn, it’s not just kind of like checkboxes. And the patients are much more complex.

1: Mmm, right. And there’s a lotta things that are not just you have this diagnosis; this is the treatment. I mean what medical school stresses, right, is shared decision making. You have this going on, and there’s several different paths we can take. And then having that shared discussion as far as these are the positives and negatives of this treatment, of this pathway. These are positive and negatives of this one, and then kind of feeling out what does the patient wanna do. And I feel like as a patient, if you just kinda saw or were given one answer or just even like multiple answers, like, hey. Pick one that you wanna choose. Here’s some pre-determined positive and negatives that you can read them, and click on what you want, that removes a lot of the personal decisions that come into healthcare.

3: I agree. I think from a patient’s perspective, I mean healthcare is very personal. You know, there’s, like, we talked about stress throughout. You know, there is—maybe I wanna know more about my symptoms or how long it’s gonna be or how I might affect other people with strep throat. You know, it’s strep throat. I mean year there’s the diagnosis, and there’s a treatment, but the conversation between the physician and a human physician, it’s very personable; it’s very—the conversation is sort of like malleable. You know, it changes based on, like, patient to patient to patient. Even if you have strep throat and you talk about it five times, it’s not gonna be the same conversation. You know, each patient is gonna be different. They’re gonna wanna know different things. So, I think I’m—I know that’s where the trend is headed, you know, AI and sort of, like, automating things, making things go a little bit faster. They’re trying to make it more efficient, but I think it’s gonna be very difficult from a patient’s perspective to speak with a machine in terms of their own healthcare.

Moderator: So, both what you said, and then I think I asked ‘cause one of you mentioned cues, like, sometimes the physician—are you both getting at the same thing there? The idea that, well, the physician might interact with the patient, might see things which would encourage additional questions that you’re saying the machine won’t be able to ask?

2: Yeah. Well, it’s like if you go to a pain clinic or some sort of places like that’ll say, “Do you have a numbness or tingling going down your arm?” Do you have this, this, this, and honestly, a lotta people will just check everything ‘cause it’s hard to—you wanna be able to ask somebody, well, what do you mean by numbness or tingling? Do you mean that my hand is going numb?” Do you mean—or the doctor will try and clarify, well, you said this. Is this one, like, ‘cause what numbness and tingling from one person’s different from another. Sharp stabbing, aching, I mean there are things that we classify things, but they’re not necessarily the way people describe things. I feel like we describe things a little bit different than what people actually experience or think to say, so that’s why the doctor as to cue the person and be like—and a lotta times even when I go to the doctor, I feel, like, when they cue in on certain things, like, do you feel like, I don’t know, asthma or something? Like, they’ll cue me on certain things. I’ll be, like, okay. Yes. I have shortness of breath when this happens, or this is what you mean by short of breath. So.

3: I agree. I think it’s very different patient to patient. Just things like review of systems, you know, or even physical exams, depending on who you talk to, they might describe things differently. An example today, this morning I was in ICU, and this patient thought he could have moved his legs up. He was in the bed, and then the doctor put his legs up, and then, like, said “Push against it,” and he was able to push against it. And everything that can describe it, he’s like, he was able to put his legs up, but in his head, he thought he couldn’t, you know, do it as normal as possible. But in terms of like, you know, upper extremity, lower extremity strength, and everything, that’s totally fine. So, that’s the thing with, like, a virtual AI, you know, computer system or a physician, if you speak to it, they’re not gonna get things like that that I think a physician would.

Moderator: Okay. What about you as the patient, so 1 talked about shared decision making, do you envision being able to ask additional questions? Not just talk to it, like we might say, Alexa, what’s the temperature today? Could you envision coming with follow-up questions?

1: You would hope so. I don’t think it—I don’t think it’s at all feasible if you don’t have the ability to ask follow-up questions. Like, without follow-up questions, or kind of a flexibility or malleability to it, it just becomes you type a couple symptoms into Web MD, and it gives you a diagnosis. If you need some kind of factor to it that makes it more interactive, and that you can ask personal things, and that you can get certain responses when you have other questions.

2: You know, what I just thought of is, like, if I broke my leg, and the machine’s tellin’ me things, then I wanna know if I go next week on my trip, is that fine. There’s not really a right or wrong answer. It’s more, okay, well, how is the pain? Do you think you’re gonna be able to walk this far? What kind of terrain it’s gonna be? And there’s no robot that could really be able to think through all those different things ‘cause those are not—you know, it’s into more abstract kinda questions.

Moderator: Yeah.

3: So, it’s like a lot of the times I feel like the physician/patient conversation sort of creates the diagram for a patient’s questions. You know, so, like, I would think it would be a little difficult for a patient to sort of come up, you know, or direct the conversation a certain way if it’s sort of like, kinda checklist after checklist after checklist rather than an actual conversation.

Moderator: Hmm. So, do you see any advantages to a virtual PC?

1: I was just thinking about that when you guys made your last points. The idea of pigeonholing, so as a physician, when you see something over and over and over again, particularly, so I’m going into pediatrics, right. In the winter months, you see so much bronchiolitis, so much bronchiolitis, so much bronchiolitis that you kind of—once you hear certain symptoms you kind of hone in and ask questions along that same line of thinking. And that can make things more efficient, that can make things easier for the patients, that can make things more comfortable for the patient, but that can vary occasionally, kind of limit your scope of thinking. Whereas a computer algorithm, I could see that it take a lot longer, that it ask every single review system, do you have tingling in your fingers even if you show up with a cold, right? Or do you have blurry vision when you just broke your toe, right. That it asks more questions than probably need to be asked, but that it might catch some of the more obscure diagnoses or things that kind of are frequently missed from a normal human physician perspective.

Moderator: But what about from the patient perspective? Advantages from the patient perspective, is that an advantage?

1: So, then the patient perspective, I mean if you’ve ever, like, had a missed diagnosis, then you can—you might, depending on what the data ends up showing, that you might end up being more confident in that diagnosis or that you get referred to some subspecialist, hey, you need this echocardiogram, or you need this subspecialist test because there’s a slight chance that you might have this, and then it catches that. Okay, well, then, obviously, you’re gonna be a lot happier as a patient.

Moderator: So, if I hear you right then, you think there could actually be an increased accuracy—

1: Yeah.

Moderator: --for, I mean the patients actually dealing with this big computer, right?

1: Yeah.

Moderator: You see that as a plus? Okay.

2: So, I agree with the accuracy, actually. Just putting on, like, a physician hat for just a second.

Moderator: Yeah.

2: In radiology, actually, it was a big fear that AI was gonna take over and all these radiologists would not have a job. In fact, it affected the specialty so much that we actually had a decrease in applicant pool, like, 5, 6, 7 years ago. It was not doc competitive. You know, they had a lot of applicants actually come in from Caribbean schools, or international schools because of that fear. You know, Watson was coming out, and it was able to actually detect x-rays, like, close to perfect, you know, pneumos, lung masses, all that stuff. And so, that was like a big fear that they had. And moving forward, sort of more so as a patient hat now, I mean I can see advantages in, like, saving time. You know, if it is a virtual physician, can we access it with our computer? Can we access it from home? You know, do we need to drive? Probably not. Money, you know, I would assume, like, if something’s automated, it would cost less than seeing a human person, you know. Maybe insurance are more willing to pay for this virtual person rather than fork over maybe four or five times more for an actual physician. So, I mean there’s advantages in both worlds I view right there.

Moderator: Mmm hmm.

1: And you mentioned the time, I think that’s a huge thing in medicine. So much of medicine is based on access, and you see so many people that—I mean obviously, most family offices work 9 to 5, 8 to 6, whatever it is. People work during those times, and there’s tons and tons of people that can’t take that time off of work to go to their doctor. And so, if you can do it on your lunch break, and go on the computer—if you can do it at home at 9 pm on your computer, that gives a lotta benefit and touches—gives those people more healthcare touches and more access to the healthcare system than they would have had previously. So, I see that as a significant advantage there.

Moderator: Others?

2: So, if it was a virtual reality that was, like an extension of the physician, then I could see a lotta benefits. If I went to a doctor’s office, and the doctor diagnosed with something, like, some kind of allergy or something, and then he left the room and the machine came on and started telling me all about the allergy and taught me everything that the doctor might forget, or not even forget but just doesn’t have time to teach, that would be nice. Like, I would actually like that, and like told me all the different things I could do at home. It’d be very thorough, but I would still—I would not want—I don’t think the virtual reality would be beneficial all by itself. I still feel like I would need to at least see the doc or something. And even over the phone, or I mean over the computer, I don’t guess I really understand how that would work. Would you be typing in your own symptoms ‘cause then that’s essentially just Wed MD. Just type in I got cough, and then they’re doin’—

1: Well, of course, it has to have some kind of flexibility, right? Ask you follow-up questions, or you have the opportunity to ask follow-up questions.

2: I think it depends on, like, yeah. So, is this—I guess—

3: Well, it depends on, like, the algorithm that is set up, too. You know, like, what ROS is gonna initiate the same review of systems? You know what I mean? Like, it’s just—

1: Well, is this a machine or is this a virtual reality?

M: I think it depends.

Moderator: It’s really for you guys to—

1: Open discussion.

Moderator: --envision. But I’m curious a little bit about what you said. So, you describe it as being kind of an extension.

2: I think it’d be great. I think it’s gonna be an extension. I think—the same thing as how it works in all the other fields, too. Even automation in machine, like, factories and whatnot. Yes, there’s less factory workers, but they’re more just extensions. They do the mundane tasks. The PCPs might become less but become more just extensions. They’ll still be the PCPs behind the scenes, and that’s what I would enjoy. If I came to a doctor’s office and instead of typing in on the ma—or typing it in on my spreadsheet, like, you know, the piece of paper saying what my symptoms were. If I talk to the machine and the machine kind of came up with the top four diagnoses, and the doctor came in the room after that, I would like that. Then I’d be, you know—

Moderator: Okay. So, both before and after. So, now you’ve described—

2: Yeah. I could see—

Moderator: --both scenarios. Okay.

2: Either way would be fine with me, but I still feel like I would need some kinda contact otherwise I’m—I don’t feel like I would be getting the good care.

Moderator: Okay.

1: So, kind of where you mentioned the extension makes me think of, like, how do you build something like this, right? You end up building some kind of algorithm and have it take all these factors in consideration, then you test it, right. And then you have human physicians check it, right. So, then anytime the computer makes a diagnosis or prescribes a treatment or whatever, then a physician would look at what the computer’s doing and say, okay. Yes. That’s right, or no, it’s not right. Maybe there’s something we can tweak and improve or some component of a learning algorithm like Watson to where it just consistently improves and improves and improves. And then you might end up reaching to a point where it becomes better than the physician, more accurate, or whatever it may be, which brings the next thought as well. Is kind of the perception around—the public perception around self-driving cars. So, if you made the AI physician better than human physicians, you’re still likely gonna have some error rate whether it’s .01 percent, 1 percent, you’re gonna have some error rate. Like, nothing is going to be perfect, and even if—I don’t think you could necessarily reach a point to where it’s perfect ‘cause the human body is the human body. It’s an imperfect system. Would the public accept using something that is not perfect? I mean if you think about self-driving vehicles, they are incredibly better, like, statistically than human drivers already. But the public can’t rally around that because when there’s an accident, then it’s the computer system’s fault. The computer system’s not perfect. We can use it. But when there’s human drivers, it’s okay, it’s human error; it’s natural; it’s kind of the way it’s always been, right. When there’s human doctors, people know that doctors will occasionally make mistakes or make the wrong diagnosis, but they’re human, and there’s kind of a line of thinking there. Those kinda things. If you introduce a computer and it misses a kid with meningitis, and the kid goes home and passes away, there’s public outcry about that. And we can’t use that anymore.

Moderator: And you think that that would be worse—it would be worse repercussions to machine failure than to human failure? If a physician misses a diagnosis, and a patient dies, will the outcry be less, or will it be the same?

3: So, I think it would be—what we’re talking about right now actually happened in the field over at radiology. But like, exactly what you said, would the whole using AI as an extension to do your job better, you know. I mean right now AI isn’t in the computer systems. Like, you don’t use Watson to diagnosis an x-ray or CT scan or MRI, but the whole field is saying, when it is out there, they’re gonna go ahead and use it to sort of backup the physician’s decision. And one of the big ideas is sort of what R just talked about with a physician, and you just said a physician making a mistake verses a computer making a mistake. So, and it’s really interesting ‘cause I actually talked about this during my interview track, so it’s worse, like sort of the general consensus is it’s worse if a computer makes the mistake because then the idea is, well, who do you sue? Who’s fault is it?

1: Yeah.

3: You know, and that’s a big thing. Whereas, like, if the physician makes the mistake, I guess there’s always that idea of, like, okay, the physician has medical school, plus residency, plus fellowship, plus sort of a stake in this.

Moderator: Yeah.

3: You know, their own sort of pocket is involved in this. And then that’s why I think—I don’t know if there’s studies done on this, but sort of the general consensus is patients want physicians to diagnosis things on images because they have a stake in it versus a computer.

2: You know, what would make it nicer, you go to the PCP office, and then you’re in the room, and the machines typing up your entire thing as you’re goin’ through, and then if you ask a question and the doctor doesn’t know the answer, and instead of just going, I don’t know, he could be, like, what is this person’s health insurance, and what will the charges be, and the machine automatically—you ask Alexa, what type of insurance, what would be the copay for his visit today, if the patient wants to know.

Moderator: So, it would be right there at your fingertips.

2: Yeah. And so, I think, somewhat, that would be a nice extension, too. If I was in the room, and essentially got two doctors, basically, ‘cause you got a machine and the doc, that would be another cool thing. Once again, I just feel like if it is a PCP, I feel like most people go to the PCP for the actual connection, talking to a person versus every—‘cause all the other special, like, subspecialties are different. But I feel like PCP, like, most of the stuff I could go to the pharmacy and theoretically look around and pick stuff out and take care of myself, but you always just wanna go to the doc to—and then what if there’s a sensitive subject? So, if somebody got raped or something like that, I couldn’t imagine talking to a machine about that or—

Moderator: So, now you’re actually coming to the next question, ‘cause we talked about possible advantages. And you mentioned a lot of things. You mentioned accuracy, you mentioned, you know, the convenience, time, and time of day, and better access, but what about some of the drawbacks? I think that’s what you were starting to—and can you imagine situations where you absolutely would not want to consult a virtual PCP?

2: Well, I think difficult topics, difficult situations, more psych-related issues, things that you actually just wanna talk to a person about, you lose the relatability ‘cause a lotta times even docs they are very relatable people, and they have deaths within their own family. So, there’s something you can relate to. I mean those are the big things to me that—

3: Emergent things, you know, things that you’re very worried about and you need to know really quickly where you want a person to see you rather than going through a checklist to finally find out, oh, you might have a brain bleed or something like that. You know, so falls, traumas, I would, you know, definitely want a physician.

1: Yeah. And I mean so much of medicine is not medicine. It’s being a person, being a listener, being somebody, someone can talk to, so you think about the depression, anxiety, the mental health issues, the social situations—somebody’s been raped, somebody had a death in the family. I think those are so prevalent in society, and so many people talk to their PCP about depression, anxiety, medication counseling, and what do I need to do. I don’t know, personally, I could never imagine going on a computer and thoroughly discussing what needs to be discussed in the field of depression, anxiety. Sure, I could feel like, yes, I feel like I’m experiencing depression. Type in I’m experiencing depression. It asks me are you losing sleep? I say, yes. Are you losing—are you not hanging out with family and friends? I say, yes. All because I would only do that if I felt like the end is, like, I wanna be on medication, and that it’s gonna give me medication or whatever it is. But it’s not a human connection, which needs to—I feel needs to be there in that situation to kinda discuss all the things surrounding a diagnosis like that.

2: And where’s the data being stored, privacy?

3: Oh, absolutely. Yeah. That’s a huge thing. And ‘cause there’s some things where anything you say into your computer, you know, the data is now stored somewhere. It’s connected to your name. What’s gonna happen to it.

1: And sold to somebody.

3: Exactly. And especially like you said, with, like, depression issues and even, like, these things can affect your job, your livelihood. You know, things like a computer versus a human might not understand you might be on, like, suicide watch all of a sudden. You know, when maybe that’s not the case. Maybe that’s just what the algorithm put you towards, and then all of a sudden, it’s on, like, a permanent record, which could be troubling.

1: Or you have somebody showing up to your house. Hey, you need to be watched.

3: Yeah.

1: And make sure you don’t kill yourself. You just, like, want some antidepressants [laughs].

Moderator: So, the privacy issue or the data storage issue, would you be less concerned about that if you’re just depending on you have a sore throat. You wanna make sure—give your symptoms and see do I need antibiotics or not. Are you less concerned there or is it the same concern regardless of what your complaint might be or concern?

3: Me, personally, just looking at—I mean as a patient—looking at what’s going on in the world, I’d be just as concerned. You know, if it was something dealing with, I don’t know, you know, depression that’s sensitive versus like a sore throat. It’s the data’s out there; the data’s, you know, it could be sort of sold; it could be hacked; it could be taken into a wrong direction. I mean a sore throat can turn into, I don’t know, maybe something like esophageal cancer. Who knows, you know. So, I would actually be as concerned, you know, even if it not that private of a subject.

1: I think if I weren’t in the medical field, I wouldn’t be concerned about the sore throat.

3: [Laughing] yeah.

1: ‘Cause I identify the sore throat with just being a sore throat and not the possibility of being something else but knowing that there’s the possibility of something else when you think something is nothing, you don’t necessarily wanna be throwing data out there.

2: But I’d also—how do a know a machine is doing what it’s prone to—hack? Like what if somebody actually hacked the machine to do the wrong thing?

3: Yeah. Right.

Moderator: So, it’s giving you the wrong information.

2: But you just never, like, so if you’re saying, like, a virtual reality, like it was an actually, like, someone could change the algorithm anytime or any kinda mistake could be put in where someone put in the wrong numbers. I’m not just saying if someone specifically hacks it, yeah, somebody could call something very detrimental and maybe no one would pick up on it for years ‘cause who’s gonna be goin’ through all those stores of data? And that’s the thing, is like, even if you had the machine see the patient or something, and okay, doc just looked over, I just feel like it’s a lotta data to be going through. Like, how are you gonna be able to see every single thing that we mentioned? Are you gonna read every conversation?

Moderator: So, are you saying that these things could be subtle so that they wouldn’t get picked up in, like, regular quality checks?

2: Yeah. I mean—

Moderator: Like tests?

2: There could be all kinds of stuff that are missed, yeah. And so, the machine’s saying one thing or picking up on one thing, but the reality is different, and then so that’s like the mistake, but find one, but if it was the other way around where somebody on purpose was, like, a bad computer IT guy wants to get revenge and puts some bad quote in there, and nobody realizes for while that would be pretty detrimental.

1: I, honestly, think I’m a little less concerned about the data situation. I mean, like, as it is right now, like, all of us have patient data in EPIC, and there’s not a whole lot we can do about it because that’s what we use. Like, EMRs is the way healthcare functions, so it’s gonna be somewhere electronically regardless of whether it’s a virtual PCP or not.

Moderator: Yup.

3: But, like, kinda counter argument to that is like we don’t have any malicious intent. I could see someone who does—

1: Patients don’t know that.

3: Right. Well, like, in terms of data, like, hacking, or like, just obtaining, you know, ‘cause like, EMRs, I mean what do they have? Soc, birthdate, name. Well, you can steal identities fairly quickly, you know, with that. So, that’s, I mean, I don’t know. I think, like, that’s a little bit kind of concerning if it does get hacked.

2: And then just relatability things, you know, what color would the machine be? What kinda hands would it have? Could you touch it?

Moderator: Yeah.

2: Is it like Apple? I’m just saying, all those different things, a lotta patients they want somebody that speaks the same language or has the same touch. That’s actually a benefit. You could have a machine in multiple languages, and so it could be—

Moderator: It could communicate with anyone.

2: That’s one of the benefits, yeah.

1: It could understand cultural concerns.

2: Maybe, yeah.

Moderator: So, how—we talk a little bit more—come down to kind of the nitty-gritty of how this might work in practice, how you would envision it? Like, you’re already talking about what would it look like? Would it have a feel to it? What about transferring your data, biometric data—blood pressure, or blood tests?

2: I think that’s what’s gonna end up happening, and it already does. I mean the nurses put in the numbers, but if a machine did this, and then took the number, and then did this, took the number, I mean I would be perfectly fine with that.

Moderator: Okay.

2: Like I said, I feel like if it’s an extension, it would be great. Other subjective things might be harder for it to actually access. How would it tell if a patient’s anxious? How would it tell if a patient is sweating or in an extreme amount of pain? I don’t think a machine could actually look at me and be like, this person’s in a lotta pain or a little pain.

Moderator: So, you’re saying that it would be the visual that would be missing?

2: Oh, yeah. Some of the subject, yeah, visualize.

Moderator: Kind of what you were getting at, the personal side of it.

2: Yeah. A little.

1: So, I kind of envision it one of two ways. Like, right, ‘cause one of the big components that you need to address is kind of the physical exam, right. How do you assess the body from a tactile and those kind of things, and how do you listen to chest and lungs? I think it’s done one of two ways. It either becomes, like, how telemedicine works where physicians on a computer and kinda wheels in, and then there’s a nurse that does the physical exam, and says what she’s hearing or that there’s a stethoscope that is attached to the computer so that somebody can be listening, but somebody has to place it, or then you just have to get to the, like, an interesting methods, and it holds out a stethoscope, like, automatically. And not having a person there, then the person has to put their chest up against it.

Moderator: Right.

1: Or that it just has sensors and finds your chest and does all those things. I think that would be kinda creepy, but.

Moderator: Yeah.

2: How ‘bout a hernia exam?

1: Yeah.

2: That would be difficult.

1: If a robot pushed a little hard.

3: Yeah. I would—I can’t even imagine sort of like an AI technology that doesn’t have some sort of physician behind it. You know, like, what you guys were saying, like, wheeling in a physician. You know, that’s still a person that’s gonna be in the room, you know, that can, like, dictate what to do or how to do it. The way I see it is, like, the whole extension part. You know, helping a physician, like, things like biometric vitals, you know. Maybe even, like, review of systems, like, creating algorithms for that. And like you said, 1, like I can’t even imagine how, like, physical exam would be—like that would—you need a person I feel like. I don’t think there’s any way you can do it at this day and age without a person.

1: At this point, for sure.

3: Yeah.

1: You need a person. I mean you envision, like, sci-fi movies where you just get scanned—

3: Yeah.

1: --what’s wrong with you, right? I mean I don’t know that much about technology and advancements, but I don’t think we’re there yet.

2: I could see maybe a little procedures becoming automated or somethin’ like that. Like, a mole removal, but—

1: Oh, yeah. Absolutely.

2: --but depends on how many PCPs do it, but if a machine just came in and zoomed in on it, and then pulled it off, or plucked it, or I guess something like that, or cut the nails, or simple procedures. I couldn’t imagine doing needles. I still feel like that might be a little bit hard, but a pap smear, yeah, I could see maybe that.

3: A pap smear could happen.

1: You never want a pap smear from a robot [group laughter].

2: I’m just saying, like, some of those, the more routine things. So, like, I bet—I could imagine a physical could be something the machine could do ‘cause it’s not really something you need to diagnosis. Just puttin’ in a bunch a data. You’re telling, like, before you go in for work, like, a worker’s physical, where you went for work, you’re just telling the doctor what’s, you know, your past history and all that. There’s not too much to gain from it, so I feel like a machine could take over that part.

Moderator: Could do that. Okay.

2: Yeah.

Moderator: What about the piece that 1 talked about before. The idea of the patient sharing in the decision making, or even ask—one of you talked about, well, what if I don’t understand? I wanna ask more questions to be able to do that, do you envision that as being part of this? Or the education piece, so you talked about that you finish your exam, and then the physician puts you with the machine that can then say this is what you need to do. Would that be good? You would be okay with that?

2: I would love that. They already do that at immunology, so they tell you what allergies you have, and then they’ll leave the room, and then they’ll have the machine play everything for ya to make sure that they don’t miss anything about the allergies. How do you use the epi pen? How do this and that. How to write down your, you know, make, like, your allergy list, like, things that trigger stuff. And I feel like that would be great. I would love that ‘cause I’m, honestly, a lotta times when the docs get to that point, they just rush through it, and it’s kind of overwhelming. And so, it would be nice if the machine was, like, this is what you have. This is, like, the things that kinda led up to it ‘cause a lotta times that’s what we do. We just go home and then we search back up on Wed MD to confirm, so it’d be nice to have that right done in the room, told me exactly, told me home remedies that I could do, told me what medications to pick up. If it listed all the local pharmacies, where was the cheapest option ‘cause I would imagine AI would be able to do that.

Moderator: Do you envision being able to access that again once you’re home, and maybe you wanna hear it again?

2: Oh, yeah.

Moderator: Or you wanna, so that would be—

2: That would be, actually, really nice. Yeah.

Moderator: Okay.

2: Yeah. I think that would be really nice.

3: I think if we’re gonna go into sort of the realm of, like, can we program this AI into, like, having conversational skills that are sort of really in depth or, like, sort of different aspects of a certain thing that can come up, it’s gonna take a lotta time. You know, a lotta time, a lotta programming, probably like, years, you know, just so. I mean I think it can eventually be done. You know, like, to be honest. Like, have a machine that can hit 95 or 96 aspects of, like, a certain issue or certain questions or certain conversations that come up. But I don’t think it’s gonna, like, be anything like month-wise. I honestly think it’s probably years or maybe even decades.

Moderator: Hmm.

3: Yeah.

2: But it would be really cool if a doc told you, you had a lung mass or something, and the machine came in and, like, showed you your CT scan and gave you, like, a 3D image, and then came in all the way to the lung mass, and then switched views to, like, show you on a cellular level. Obviously not your cellular, but, like, a diagram. I feel like it’d be really cool. I feel like I’d be much more involved in the entire process. Like, I would be learning a lot, so at my own level.

Moderator: You’re talking about really enhancing the patient engagement.

2: Yeah. I think that’s what patients want. You wanna be educated. That’s why you go to the doctor’s office. You wanna learn about what you have, what’s going on, and then how to take care of yourself [15.44]. That’s what I would like a lot.

Moderator: Are you saying that that’s not possible today, in a regular physician’s office?

2: I don’t think it’s gotten to that point yet, no. I mean maybe with some of the routine stuff. This is what we always do. You find out what the person has, whatever, and then the end of EPIC you click on cough, and then it’ll print off a generic piece of paper that says, cough. What to do if you have a cough and the cough gets too serious, call 911, blah, blah, blah. It’s very generic.

1: Yeah.

2: And it’ll be the same cough for somebody who’s coughin’ up blood, someone who’s coughin’ from asthma, someone who’s coughin’—and so, it would be nice if, like, the AI came in, and like, took exactly what the doctor said and was able to formulate something specific to you, you know, not generic, not just the standard machine jargon, specifically to you.

Moderator: But so, that’s again, based on what a living physician has.

2: Yeah.

Moderator: Okay.

3: And that’s what I was thinking. It’s sort of, like, if you wanna enhance a patient’s experience, and we’re talking about, I think we can all agree, like, there’s a physician there at some point within the interaction. Like, a live person versus, like, okay, if they’re solely a computer and we’re not talkin’ about any physicians or any human beings, that’s a different story. That I don’t think we are, but enhancement, I think we’re kinda there. You know, it just depends on how you set up your practice, how you set up your EMR, how you set your health system. You know, so I mean in California, Kaiser Permanente, it’s one of the largest health systems there. And like, they are very good at mining data, and very, very—

Moderator: And a lot of it.

2: --and a lot of it they set up their EMR like that ‘cause, like, they publish tons of papers and tons of papers because of that. So, I can see, like, you know, depending on how big a community is, you’re setting up your system in a certain way, you know.

1: I guess one place it could work is, like, we always talk about rural physicians, so like a rural area, why not. You know, like, it’s better than nothing. That’s the thing, and so if it’s like rural Montana or something. It still costs—if I was in rural Montana, so it costs a lot to get the AI out there. Pay for the AI, all that kinda stuff, so I don’t feel like it’s still financially be feasible—

2: But depends how you use the AI, right. If it’s just, like, a computer, you just go on a computer program and you use it, and then use, like, a nurse or accessory healthcare professional to do a physical exam and show the—write—type in the physical findings.

1: So, in a rural setting, I think it would be pretty beneficial ‘cause you hear about there’s not enough docs in those areas, people are movin’ out, all that. So, I think, yeah, it would—that would work out nice. A nurse came in or something.

3: Yeah. But then if we’re gonna put that aspect in it, then you gotta take out some of the advantages, right. Like time. You can’t do it from your house anymore you have to go to a certain place and see a nurse. Money. A nurse has to get paid you know. Someone has to get better. So, it’s sort of like what’s the middle ground. You know, how much—

1: Yeah.

3: --people, how much AI.

1: Yeah. I mean it would be nice if, like, you live in a really rural area, and there are no doctors in your town, and you have to drive 2, 3 hours to see a doctor, and then there’s a 2-week wait, 4-week wait, whatever it is, it would be nice just like almost as a screening tool to see this virtual physician. And like, should I be concerned about these things? At least to get some sense even if you don’t believe it to be a 100% accurate, it gives you some idea of how important is this? Like, do I need to take off work to go see that doctor, or do I not need to take off work? Like.

Moderator: So, could this be a reality?

1: I think it will happen to a certain extent. I don’t see it fully happening as fully replacing the PCP or the role of the PCP, but I think as a screening tool, as a quick diagnosis for, like, people who have strep throat, can I get a quick throat swab? Those sort of things, but I don’t think fully as an independent PCP.

Moderator: But you still see a possible role for it.

1: Yeah.

2: I think a 100% possible. I think AI will be in every field, so there’s not a doubt that it will be in medicine. It already is in medicine, so it’d just be how much, and like I said, I think similar of just be an extension even like triage. It might help with a post diagnosis or pre, but I still think they’ll be people contact. Then in rural settings, they might just help with, once again, I guess that’d be also triage ‘cause you’re just putting in your diagnosis, wanting to see if you should drive to the nearest place, or so. But it’ll definitely be—no, there’s no doubt about it.

3: I think it, like I said, it’ll be an extension. I don’t actually ever think it’ll every replace the actual humans unless, you know, there’s some dire situation. Not a PCP, but surgeons. I know some surgeons right now they’re working on doing surgery remotely, you know, where there aren’t surgeons using the robots, and if they’re like in Grand Rapids or something doing the surgery in the upper peninsula. So, but that’s still, there’s a physician component to it, and I think that’s where it’s going. I don’t every foresee it, like, replacing the physician due to all the things that we just talked about.

Moderator: Are there any medical specialties, again, try and think as a patient, if you can, where you could say, yeah, this would almost even be better for me, to start with this.

2: Well, then you go to radiology.

3: Radiology?

2: As a cornerstone, but it’ll also be just an extension of radiology. I mean back in the day, ultrasound still needs to be done by a human, so it needs the movement, all that kinda stuff. They’ll still come out with newer imaging that might require human intervention, and then certain things you might need stat, and a machine will still take time to read things. A human might just immediately be able to go no, this is, you know, or you know, they’ll still be, once again, elements of a physician needed, but that’s probably the biggest field where you will see stuff. I would also think pathology. I thought about that.

1: Yeah. I would also say pathology big one. I don’t think it can fully replace any field, and so I don’t see it fully replacing any field. I think there are a good number of fields where it can be used as kind of the initial, and even then, essentially, require less physicians to treat then the same number of patients. So, like, chest x-rays—simple chest x-rays, even a lot of times don’t necessarily need the radiologist to read when it’s a simple pneumonia, and you’re gonna treat the pneumonia, and then any physician who’s had any training identifies that is a pneumonia, and the kid looks sick, and so you give antibiotics. But you’re never gonna replace the complex things, so one field that came to mind is something, like, cardiology, like, outpatient cardiology where, at least in pediatrics, you get your four extremity blood pressures, you might get an echo, and that’s kinda your—can be an initial starting point and can at least screen the severity of things or screen the importance of things, and might save kids from needing to see the pediatric cardiologist per se.

Moderator: Mmm.

3: Radiology, probably, just because of the amount of image—I mean it’s all images other than, like, the procedures that we do with interventional and biopsies. Maybe GI, you know, in GI a little bit is going towards that, like, colonoscopies, you know, that’s sort of a screening tool, but I mean it’s not recommended yet, but slowly—what’s that called? They’re using the news, like, CT scan thing.

2: Oh, yeah.

3: Like, you guys don’t wanna talk about, like, they just take the images and then, like, a radiologist reads it rather than, like, a GI person. But then the flip side is, if there’s something abnormal, a GI person has to go in and actually do the colonoscopy—

Moderator: Anyway.

3: Anyway.

Moderator: Yeah.

3: So, I mean I agree with 1, I don’t think there’s gonna be any, like, one profession where it’s, like, completely taken over. I think every profession is gonna probably use AI as, like, and extension of themselves just to make their profession a lot better as well as the patient’s.

2: If we were to teach AI how to do physical exams, I bet nurses would be like, teach ‘em how to change diapers, and something, like, more not pleasant tasks of being in medicine. So, I feel like that would be a higher [laughs] priority than—

3: There’s those robots at, like, MIT.

2: That change diapers?.

3: No. They’ll be—they’ll, like, walk like dogs, and you mentioned stuff like that.

Moderator: Oh, yeah, yeah, yeah.

2: Yeah. So, I mean they probably have something they could do stuff with hand, you know.

Moderator: When you think about this a possible reality, do you foresee patients refusing? Absolutely refusing to have anything to do, or do you think that will be just—that will change with time anyway that we’ll become so accustomed to, as you say, AI is already everywhere.

1: I think both of those things are true. I think anytime you initiate something new or something, like, there’s people that refuse. And particularly for the sensitive topics, maybe, or people that just feel like they want somebody to talk to, that they’re gonna refuse. That’ll be fine, but then eventually as kind of generations grow, and it just becomes the norm, everybody’s gonna use that.

2: Right. That’ll be the nice thing if we allow them to refuse, but you call up comcast or any of those places, you can’t refuse the machine. You’ll sit there.

3: They keep pressing zero [group laughter].

2: It’s, like, they’re starting to now stop allowing you to put zero, and it’ll keep asking you questions. It’ll be, like what’s your account detail. What’s your this and that. So, if they do, do a refusal, it’ll be nice in the beginning.

3: And I almost wonder, like, sort of like, what is the rate of, like, them changing services at that point? Like, if you can, you know, the frustration just building up, like, and you can’t talk to a patient, I mean a person, I guess. Like, would you like, okay, screw Comcast and just doing AT&T.

1: I’ve lost so many internet providers.

3: But that’s why I talk to people.

2: Yeah. It’ll create a new market, like, and now they are cash-based primary care, so you miss the insurance, cash-base PCP where you bypass the insurance, you bypass going to a machine, and you’re actually gonna see the human.

3: There’s those—I don’t know if it’s cash-based, but you know, those like, it’s part of Spectrum, but you pay, like, kind of like, a fee every month or whatever, and you get, like, access to a—do you know what I’m talking about? There’s a—

1: It’s like concierge medicine.

2: Yeah. Yeah.

Moderator: Yeah.

3: Yeah. So, those are there—

1: Concierge primary care.

3: Yeah. And you get, like quicker access to a PCP, and you pay extra for it. Yeah. In terms of like, a patient sort of saying, “No. I wanna talk to a physician,” I think, like, that is a fear. I mean not a fear, but that is a reality. That’s absolutely gonna happen to me. What’s healthcare now, like 20% of our GDP or something like that? So, like, you know, even though I know you’re asking us, like, put our patient head on, the good portion of the population are, like, somewhere, some way, you know, involved in the medical field I feel like, you know. Like, maybe it’s low. Maybe it’s 10%, 5%, whatever, but that’s still, like, a good amount, you know, a good number. So, I think, like, a lot of the times we’re just gonna think, like, oh, we know more than this computer. I need to talk to a person. You know, and I think with Comcast or stuff, like, that, yeah, frustration hits; we change it, but with our own health, you know, that’s a different story. What are you gonna do? Yeah.

2: You can log in on MyHealth—

3: [Laughing] oh, right.

2: Frustrations.

Moderator: Yeah. Those were the questions that we had for you, and do you have any other thoughts or comments that you wanna share?

3: Can we ask about your thoughts, or is that –

Moderator: Oh, personally I think that the three of you have echoed a lot of my own feelings. Um, I think it’s hard – it’s hard for me as an individual to envision this as the only type of healthcare encounter in a primary care setting. So I think you’ve described a lot of possibilities that will probably, someday, be there.

3: I almost think that when you asked that question about specialties, it sort of dawned on me. It would be the hardest, I feel like, to do in primary care. It would be the hardest to do in family medicine, internal medicine, peds, you know?

1: Yeah.

3: Just because those are the specialties which sort of like, take care of the patient, and like, dictate it towards other specialties if needed. So, I don’t know, it’s kind of interesting to me where like, we’re taking about AI with PCPs because I feel like that would be the hardest to implement.

1: Mhm.

3: In my opinion

1: I agree with you. I think if you only take the element of a PCP as like a navigator, like, oh you have derm problem, I’ll send you to derm. You have a surgery problem, I’ll send you to surgery. If you only take that aspect, I think AI could do a fantastic job. But the problem is that’s such a minor aspect of what a PCP does.

3: Yeah.

1: I absolutely cannot imagine it replacing all of what a PCP does.

2: If we’re not talking about a PCP then – one place it will definitely go, as all new technology goes, is the Army. I mean if they all have it on the front line, kind of thing. That would be a perfect place, like if they don’t have a doc in the field or something, then you’ve got a machine. And once again, I don’t know how you’d get the data and all that stuff, but I feel like it would definitely be a good fit in the Army. For cost-reduction, using it in the field, moving it from location to location, speaks different languages, can treat multiple different types of people.

3: Yeah, they have like, their own little communities too, you know, wherever they’re stationed.

1: Yeah.

2: Yeah. But yeah, I agree with you that PCP would be hard. I don’t think it would last and take over.

3: Yeah, right.

Moderator: Yeah. Anything else? Well, thank you so much for taking this time and for sharing your thoughts.
