# Supplementary material for: Perceptions of virtual primary care physicians: A focus group study of medical and data science graduate students
Source: PLoS One. 2020 Dec 17;15(12):e0243641. doi: 10.1371/journal.pone.0243641 (PMC7745971; doi:10.1371/journal.pone.0243641)
Supplement: S1 File — (ZIP) [file pone.0243641.s002.zip › Anonymized Transcripts/vPCP Focus Group 3 anonymized.docx]

vPCP Focus Group 3

April 10^th^, 2019

Present:

Moderator

Two note-takers

Four 1^st^ year graduate students

Moderator: So, Bengt explained that what we mean here by a virtual primary care physician is, it’s a machine, right. It’s artificial intelligence, so what is your thinking, what’s your view of using a virtual primary care provider? You’re free to speak as the thoughts come.

1: Okay. So, yeah, I believe it’s, yeah, there’s—it’s a broad question and broad answers for that, so yeah, it is definitely very good I would say because sometime we—definitely while doing treatment, so anything, being a human-being we do miss, but if we give some commands to a machine, it’s like already coded everything. So, there’s no chance that it will miss. Definitely if something happens to machine, that’s okay, but like, being a human being, like, there are so many several cases by mistakenly people miss something inside the body, and something, you know. So, I think definitely it will reduce and also, yeah, like, I don’t know, like the country which I belong, we—or maybe my family, we have never seen very major surgeries or anything, but yes, like, human beings, we are attached emotionally with humans, right. Of course, while cutting, and doing else, but if it’s machine, there’s no emotion attached. So, it’s beneficial, but at the same time, pros and cons for this. So, yes, but definitely, it’s a very good thing.

Moderator: Okay. So, what I hear is that largely you’re very positive to it?

1: Yes. Very positive, of course. I think medical is, like, something—artificial intelligence actually is growing in every field, but medical is something that is actually required. Like, we can drive a car, yeah. I understand that artificial intelligence, like, it’s good in, like way cost more ‘cause it’s good, it’s beneficial, it’s required in medical.

Moderator: Mmm. Okay. Other thoughts?

2: So, in my point of view it would be concerning because machines only learn from causality, so in medicine I don’t know what types of instruments would people use to detect that? For example, a physician used to give prescription or something, but a machine only learns from causality, so if something is a cause of, like if something has a correlation, that should be the cause. The machine says that, so all of us know that correlation does not necessarily mean causation. But machines always have to make one choice, and depending on the emergency of the issue, intent, machines have to, in my point of view, machines have to also contact, maybe, that real physician about the decision that it makes because, you know, we know from certain, like, there are some rare medical conditions that happen, which have similar symptoms, maybe, with some very well-known diseases. So, in that case, machine might be wrong in its decision. That’s what I’m saying because they have to make their choices, like, they have to make their choices using some probable decisions. So, that’s what my main concern is, but on the other hand we have a physician that can be multiplied, like without any costs, and it would be, like, it would be maybe free. That would be the greatest achievement and would really speed up things. For example, a simple flu or a simple, like, simple allergy, it can detect that. And even if it were to use some vitals of the patient attend, it might provide really good insight. Then we come at the issue of privacy and other things, but I would like, to like, leave time for me colleagues.

Moderator: Okay.

3: I also kind of have my mixed opinions about it. So, for the good side is basically if you have no central system that basically can eliminate, for example, if you go to a doctor, there’s some good doctors, there’s bad doctors. But if you have a virtual physician, that’s on average, that would be like good enough, so that people generally will have, like, generally, good opinion as in they have the large system collect a lot of cases and stuff. So, they would be able to, the virtual physician would be able to give them more fair views on the treatment and stuff ‘cause sometimes you just have, like, inexperienced doctors that you just, they mess up things. But on the other side, like, disease and… mutation, or things just keep changing. Like, how updated the system can keep track of—there’s just so many, like, individual cases. Like, this might not work on this person. Like, just how much data can the virtual physician have to—just to give, like a right, fair opinion to them ‘cause it might be, for example, some doctor just—I have a very personal experience with, like these groups of patients, and they have some kind of—some very intuition by just experience. But then, since this virtual physician probably a database and how also by AI, but just how can it protect to continually changing of the diseases?

Moderator: So, are you, just so I understand, and I think you were in on this a little be, too, Seyyid. So, are you saying that the AI system would need—you seem to say validation?

2: [Inaudible].

Moderator: Maybe human validation.

2: Yeah.

Moderator: And if I’m understanding you, you’re saying that it’s not growing. It doesn’t have intuition, and it doesn’t have the experience?

3: Or how do they keep updating, like, the newest—

Moderator: Update. Okay. To keep it dynamic.

3: Yeah. It’s a little safer to, like, if for example, like, the system is very open source and, like, doctors and stuff can just keep putting information in there, like, constantly, then it might be a very robust virtual physician.

Moderator: Yeah.

3: But, like, they’ll just have to be afraid, like, people who manage it, like, well enough that it actually does what it’s supposed to do.

Moderator: Okay.

3: Exactly. You’re saying, like, oh, I didn’t get this part, and then the AI’s doing something. So, if it covers, like, a huge group, then I think it’s trustworthy, but like, how would the patients know that. Like, how trustworthy is this system.

Moderator: Okay. Thank you for clarifying that.

4: I have to learn you, so basically it depends on the situation whether there’s emergency fees or basic checkup or something like that.

Moderator: Okay.

4: So, it depends on the person. What if I’m entering it wrong, feeling of mine, so it might end up, I may get a wrong result, right?

Moderator: Mmm.

4: So, if I’m doing something wrong, which I actually I don’t know what I’m going through, so it’ll be better if I talk to a doctor and tell them what I am going through, right.

Moderator: So, okay. So, are you questioning yourself there?

4: Yes.

Moderator: So, you as the patient—

4: Yeah.

Moderator: Okay.

4: Yeah. So, if I’m not aware of what I’m going through and if I get some wrong into that, so I might enter with dealing with some other disease, really, right. So, that’s one point, and the second point I feel, I’m not too handy, like, you know, it’s rough contacting the person, it’ll be very helpful if you just talk to a machine, and know the disease in a very short span of time.

Moderator: Yeah.

4: But when it comes to a huge kind of thing, I feel it has to be evaluated by a doctor, like, to the specialist and know what you’re going through. I think that will be more safe I guess.

Moderator: Okay. So—

1: I would like to add this point.

Moderator: Yes, please.

1: So, basically, like, I think 1 ½ year ago in south part of India, like, my country—home, so we had a disease. I forgot the name. It was like a virus, and it actually, it was like spreading, and so the person who was, like, helping, like, nurses and all, they’re also getting infected.

Moderator: Oh.

1: And this one nurse, like, she served, like, she saved a girl, but she died. And that was—

Moderator: The nurse, excuse me. The nurse died.

1: The nurse died who took care—it was a very sad moment, and that time I felt like we need AI. If there would have been a virtual machine to do that—we were very sure that, okay, let’s put the machine inside the room; let’s not enter there. And you would have saved that girl, so I felt like some extent, like, in some areas, we definitely need AI.

Moderator: Such as infectious disease.

1: But yes. Infectious disease, and like, yes, and I would agree with all of them in saying that, yes, we cannot just rely on artificial virtual machines, like, definitely, each machine should be supervised with at least a physician. That we can actually reduce the number of this, but yes, it should be supervised and verified with, like, you cannot just rely on machine, definitely because as if we don’t know what’s going on and sometimes, I’m just, like, hey. I don’t know what is this. But if I say it’s, like, stone inside but it’s, like, my appendix inside, and you put the machine appendix, you would remove my appendix, but I wasn’t knowing that it’s a stone. I’m just giving example, you know, so it’s like a—‘cause we are not an expert or doctor as part of that, so we might end up saying wrong.

Moderator: So, you’ve touched on this a little bit, but what are some clear advantages? Are there any advantages that you see with a virtual PCP? Are there—you mentioned a situation where you actually think it would be preferable, right?

1: Yeah.

Moderator: Where you have an infectious disease that’s spreading, where if people could be helped—

1: With the machine.

Moderator: --with a machine rather than with another human, of course, you could save lives. Are there other advantages that you can think of?

3: Well, I think one, like, big advantage is that it’s fair because sometimes if you’re rich, you get, you might have access to better doctors, and yeah. So, basically, if you’re using the virtual physician, so basically, everyone gets access to this specific one. And so, it’s not, like, because you cannot pay that much to see this specific doctor who really could’ve—and you’re left out.

Moderator: Are you envisioning that a physician would just be—an AI physician would just be an AI physician? Could there be such a thing as a specialist like we have with human physicians, yeah.

1: Yeah.

3: If you use the computer, I would think, like—

Moderator: It is a specialist, right.

1: Yeah.

3: --it should be a specialist on everything.

Moderator: Yeah. Yeah. Very interesting idea.

3: ‘Cause unlike, for example, doctor, like, they focus on—they have so many years of school, but if you’re a computer system, you can just put all the information, you don’t have to go to, like, I don’t know, 7 years of school, and then this person—‘cause there’s so much information. But the advantages of computers, you can just put all the information in.

4: And in many cases, you won’t feel like sharing what you have, what you’re going through with another person. So, in that case, for maintaining your confidentially or secrecy. It would be better to be with the machine.

Moderator: Can you give me an example?

4: For example, if you have sexually oriented—for something like that.

Moderator: Right.

4: So, instead of—if you feel shy enough to go to a doctor and then discuss about it, maybe if you have a machine you can talk, you can tell what you’re going through.

Moderator: Okay.

4: Maybe another -- People don’t like to tell skin diseases, you know, allergies, so in such cases, it might be really helpful to talk to machines. That’s what I think.

Moderator: Other advantages?

2: It means a lot of cost in terms of maybe time costs for real physicians, so for example if it were to be a help like that—helping hand for a real physician, a person who has some questions would first go to the virtual physician, and maybe after that they would go to a real physician. It would help. On the other hand, it would maybe, like, take the job of some real physicians. That would be scary, but it would decrease costs. It would decrease time, like, time requirements for physicians because as far as I can see they always are shorthanded. They always are, and they always have to catch up with patients. And there are some patients who have lots of questions. Like, even simple questions they, like—they have the right to do so, obviously, but still like it consumes a lot of time for the physicians. That would help. On the other hand, even if were to like—even if patients were not to use it, even if only physicians were to use it before they come to the room, the virtual physician would have already given, like, it’s like a fortune telling. Like, it might be something like this, and the physician wouldn’t have to maybe ask a lot of questions and just on his professional basis, he might come to the conclusion that, okay, this is a simple disease. This can happen. This is very normal, and I don’t have to spend a lot of time with this person, and I can only explain what happens to them and don’t have to maybe ask a lot of questions. So, even if it were not to give opinions, like, maybe recipes, like, sorry. Like, even if it were to not write—

Moderator: Prescriptions? Yeah.

2: --prescriptions, to the patient, it would be able to give opinions to the physician. And sometimes it would even maybe cover places where the physician wouldn’t, like, think of—there might be some cases, who knows. It would be an advantage.

Moderator: Okay.

1: Yup. Like, maybe I’m being very optimistic, but yes, I am imaging a world where, like, yeah, we have, like, enough virtual physicians and all, and then, yeah. So, sometimes what happens, like, we have some specialist, like, in the world just one person who’s, like, the best in this particular cancer or something, and then if this case is, like, in another corner of the world, so in that case, one has to travel. Like, either person, or -- But if we have virtual machine, like, we can create a number of it, but humans we cannot generate, right. If we have one specialty, we can train, but still that efficiency, we cannot guarantee. If people are like, okay, I want that specialist to be sure that I’m getting treated well. But if it’s a machine, same company, same coding, everything the same, we can spread a number of it, and then so, we can actually—

Moderator: So, are you saying that if someone presents with perhaps a very rare condition—

1: Mmm hmm.

Moderator: --a physician in an office, a human, might not be aware—

1: Be able to reach there.

Moderator: Right.

1: On time.

Moderator: Whereas, okay.

1: But if machine, like, suppose there’s one person in the whole world who is, like, part of this particular disease. Like, suppose a cancer, and then so someone is, like—I’m like from India, and someone is sitting in America, and the doctor is in America, and I have to reach in time. It’s, like, emergency case. So, I’m actually thinking, like we had a case, like, 2, 3 years back in Delhi. There was a girl with gangrene, and she was, like, very serious condition, and like, we took her to I think —I think I don’t remember exactly where, but it was like, abroad—overseas. It took so much of time we couldn’t save that girl.

Moderator: Oh.

1: So, if it would have been a with machine that expertise -- the machines were in everywhere, like, not just one person, but similar to the expertise of that person if you have more machines spreading to all the countries. We could have covered this sort a distance, and we could have saved that girl. So, definitely, like, in these things I feel like we need it. It’s not just, like, [inaudible] or something. It’s not for, like -- but we needed artificial, virtual physicians.

Moderator: So, I hear quite a lot of advantages.

1: Yeah.

Moderator: Everything from infectious disease, getting expertise that might just be in one single place far away, talking about dealing with sensitive situations; it’s fair. It becomes a just way of treating. Everybody gets the same treatment.

4: It’s time saving, too.

Moderator: Time, and perhaps cost saving. I think you—

4: Cost saving.

Moderator: --also mentioned, so it can be efficient. Any other advantages or situations in which you would definitely see this as a better choice?

3: I guess the same as time saving, but if you have a virtual, then you can basically contact whenever you have problem. You can just, maybe sometimes if you’re just feeling a little pained, and you say, oh, you have to go all the way to the doctor and wait, then maybe just let it go. But then if you’re concerned, then you can just, like, access it really easily. Then maybe it would change the outcome ‘cause sometimes it’s an emergency.

4: Yeah. Easy access if 24-7.

Moderator: Right.

4: You could contact it anytime.

Moderator: So, that’s part of the efficiency, and it’s also, maybe we could call that convenience, right.

3: Yes.

4: Yes.

Moderator: Right, for the patient. So, what about drawbacks? Are there any negatives?

3: Continuing on the advantage of the sensitive information, I think it also can be a bad thing ‘cause what if it got hacked—the system, and then all the information leaked out. Yeah, everyone knows.

4: Yeah. Suppose there is a celebrity who is in contact with that machine, and he’s telling all his datings, and suppose that particular machine gets hacked, so all of his information if leaked, right. It’s, like, his privacy’s been leaked.

Moderator: Okay.

2: I’d be more concerned about the biological warfare in that case—

4: Yeah.

2: --because you can even target the country with this—maybe continents, maybe, who knows.

Moderator: So, that is kind of a general drawback. Can you think of situations where absolutely would not want to consult virtual?

2: I don’t know if this counts, but I would never consult a virtual physician in any of my physical—any of my sicknesses because I would think that the data, and it’s one time or another, would go into hands of the somebody—would go into hands of someone that I do not trust with. So, just, like, you cannot—so, we do trust our information with the healthcare system, and it is the same as maybe trusting your data or problems with an AI. But the thing with AI is it can make deductions from those data, and it can bring out a lot of issues about your life much faster and maybe much detailed than a normal person. Like, a person cannot go the files of each and every single person and make deductions of those persons’, maybe, medical files without being assigned with it and without having a lot of time. But a computer can do it very fast, and it would be dangerous. Still, even in the system we have our data, so that, yeah. That would be definitely—when I wouldn’t want virtual physician to exist. On the other hand, they might be biased. They can get biased very fast because if, for example, in a region, a sickness if pretty common, it gets pretty common, and the symptoms of that sickness is the same as another one.

Moderator: Uh huh. Similar you mean, but not the same.

2: Maybe even similar—even similar would be enough, actually, for an AI, so at the end it would make wrong assumptions, and it would be biased. It might even get racially biased because there are some distinct racial characteristics in their biology, and at the end, it might even get racially biased in making decisions. So, it’s pretty easy for it to get biased because it only makes deductions, like, with no ethical concerns. It doesn’t have any equality perception. It doesn’t have any—it is completely and, like, utterly—only looking at the correlations.

Moderator: Yeah. Mmm.

2: That can be added as a constraint, though. I don’t know. I don’t know how that would be.

3: Yeah. I agree with that, like, for general, common disease, like, you can trust, but then for some pretty uncommon, like, how can one trust their treatment? They diagnosis the right, yeah, the right diagnosis of the disease? How can we as patients know that they’re right about it ‘cause AI is—keeps changing and adjusting, like—

2: That is even the case for a real physician, though, yeah, right?

3: Oh, yeah.

2: Mmm hmm.

3: But ‘cause I’m, like for example, yeah, the real one, also—for example, if you go to the one that specialize in this, then you’ll be—

2: Mmm-hmm

F: --but yeah, it’s also kind of bias. But my point is like, how can you trust—‘cause then, it’s a computer. Maybe, I don’t know, sometimes face-to-face, when you talk to the doctor, then you feel—or maybe they can be trusted. Like, if you just look at a computer, like, how can you determine if—

Moderator: So, are you saying that you would feel less trust? That that’s one of the drawbacks for you, that you—

F: Yeah. Like, how can—I guess, how can they, like, for example, doctors, they have reputations of, like, the background and stuff, but how can, like, for computer, how can you, like, trust, like—

4: Ability—trust is ability to—

Moderator: Sorry?

4: How can we trust it’s ability to—

Moderator: It’s ability?

3: Yeah.

Moderator: I was wondering whether—I mean a physician, you said something so interesting. Now, a physician has a reputation, right. Where does that reputation come from? Well, he or she, is successful, has good outcomes –

4: Experience, degree.

Moderator: Experience. We look at surgeons, and we say that if they’ve done a certain number of thousands of a certain type of operation with good outcomes, those are all part of their reputation, right. So, can a virtual physician develop a reputation?

1: I think so. Like, yeah, because it’s like normal life. But I’m, like, from an engineering background, and we use various equipment. So, like, for particular, imaging or something, we say, aye, we should go for this particular brand, and this microscope because that’s something seen, and that’s a reputable one, right. Because of experience we have been using it. We are getting good results, so same we can do with virtual physicians, too. But, yeah, definitely, too, it will take time. And, yes, humans, and anyone’s life is not that we can do experiments, but at the same time, we are doing, like, stethoscope. This is also one of the artificial intelligence. Right now, we are measuring how the hardwares are going. Thermometers. It’s like the machine, we are measuring the temperatures, so we are trusting now, right. So, with the time it will get developed, definitely. The reputations as, like, the physician, same for the machines also. But, yeah, if we—if you ask me to a characterize where I should go, and where I would not go. If I’m having a very common, like, common cold or something, a fever, I would go for virtual because I know it’s like something I’m having, like, fever and sneezing, cough, cold, and all.

Moderator: Right.

1: This is very common, and I’m sure that I’ve—like, millions of datas, and I can trust because I’m trusting my recounts. It can fail at any time, but I’m driving it. So, I can trust, but if something is, like, in between, I’m confused, like, the situation where you said, you don’t know what exactly is going on, I would go for a, like, human being rather than going for a virtual physician. So, there should be, of course, option, but when it comes to the surgeries or something, I would say we can go for virtual physicians to do the certain ways of surgeries and all, so, because if you’re deciding to go to the finals, it’s definitely we will study the patient carefully. If we have enough medicine, definitely we’re gonna study where his intestine, what exactly, and all—how much you test to cut them out.

Moderator: Right.

F: So, we will look for that, and I think it’s a good way to go for because if a human being there, I think they would perform the same—human can do some mistake, I believe. But, yeah, there was, like, someone is sitting in another room and operating, the machine is doing all of the cuts, and you are just sitting here, and you’re looking what’s going on. And if you feel, okay, you can kinda stop it there right now, but you just not being over there, and you are not, like, facing any emotion, any mistakes because a human being is something that can happen to. Like, I’m not feeling well today. I’m not in good mood today. I had, like, something at family—something happened, and doctor might—‘cause they’re also human being. They can also get changed by their mood, but the machines will also be unbiased every day.

Moderator: Okay.

F: It’s just that we have to, like, every day inspect that the machine is perfectly working fine. So, I believe it’s good, but yeah.

Moderator: Any other clear drawbacks or negatives or times when you absolutely wouldn’t wanna consult it.

2: So, I wouldn’t be very optimistic about public being open to this kind of thing because, for example, there are thousands of accidents, unfortunately, every day. But when an Uber with self-driving car makes an accident or a Tesla makes an accident, it becomes national news.

Moderator: Yes.

2: At the end, this would require lots of, like, safety precautions, and it is good. It is nice, but at the end, it’s also brings some transparency issues. It brings—I don’t know. It also brings a different platform than a real physician. As you said, can a virtual physician develop a reputation? It would be down to the ground in a single mistake.

Moderator: Yeah.

2: Yeah. A real physician, it wouldn’t be like that. We would say, it’s a human. It can make a mistake. A human error. Like, that’s what they call. The machines do not have that chance. Like, they cannot make a human error, and that’s why actually I would say they need some human intervention at some point. Like, in some mild conditions maybe because, as my friends here said, I would trust a virtual physician if it is a very easy condition. Like, if it’s a very simple condition, like a cold, like a flu. I even go search internet. Like, it is simple to -- it is similar to that, actually; it is simple, but at the end, I would say, like, it’s—public is not very pretty open for this. It wouldn’t be, because they wouldn’t understand what’s going on behind it, and also, like, there are other like—there would be some people which have same concerns as me, like, as privacy concerns.

Moderator: Mmm-hmm. Privacy concerns in general or about just specific things, like, what you mentioned. That there might be—

2: Well, actually, like, maybe, maybe more evil schemes, like, if I put it, like, a machine selling my data to some—

Moderator: Oh, you mean like that.

2: Like a Cambridge Analytica, for example. That’s a pretty simple, actually, example for this. Like, they were not even selling some vital data, but that data allowed them to maybe change the results of an election, who knows. So, like, it had pretty important effect, and like, the data seemed not important at all, and at the end, it changed a lot of things. Even knowing the relationship between people changes a lot of things. Knowing their medical history, medical data, and adding them to—like, on top of each other would create a huge amount of information, and at the end it can be manipulated more severely, I would say. That would be another concern for general public, and it is a concern for me, actually.

Moderator: Yeah. Because your data might be included, also, in—

2: Of course. Of course, so no one actually prevents that. Like, no one can actually prevent that. There will always be a way to get that data from there, so a Facebook can actually block Cambridge Analytica, and Facebook can block other applications, but it would not be able to block everything. At the end, it would not be Facebook if it were to do that.

Moderator: So, we’ve talked about some advantages; we’ve talked about some disadvantages. How do you envision? How could it work in reality? If you imagine yourself approaching a machine for a consultation, how would it work?

2: I guess it would ask some similar questions as doctors do. It would ask my weight, height, other vitals, and then ask for the problem. And depending on the problem, it would maybe ask other questions, and it would provide some, like, several issues that might be happening.

Moderator: So, you envision it sort of like an algorithm, a tree, so it asks you questions, you respond, and then based on your responses, it comes with—

2: Yeah.

Moderator: --new questions.

2: Yeah. New questions, and like, it narrows down to scale the diseases, the number of diseases that I might have, and it also should take into account that I might just be healthy. And it also should take into account that I might not provide enough information. That is definitely the case that the user might not provide enough information, and a user might even lie. That would definitely happen, so it should envision this case. And at the end, it should give some diseases, and maybe probabilities for those diseases.

Moderator: Okay. So, it could say, you could have x, y, or z.

2: Mmm hmm.

Moderator: This could be. So, you describe it as kind of a—it’s almost just a question and answer. It’s like an interview. How do you others—do you see it like that?

1: Okay. I just imagine, like a three company, at least, together. So, the human being, and one machine. Like, two human being can be two patients. Like a patient with an accompanying person—

Moderator: Oh, okay.

1: --or a patient alone, but a physician, or maybe instead of having, like, multiple person from the hospital, just one at least. So, I imagine two person. If a patient is not able to enter the data, someone is there. Like, for—

Moderator: To assist?

1: --for accompanying—accompanying, yes because it was, like, it asked me to speak, but sometime my accent wont’ be, like, you know, I’m from another nationality, and I want to, like, the virtual machine is not able to recognize my voice. So, maybe that time I’m very weak or something, so my accompanying person can tell that. But if supposed I’m good, and but I might, yes, at the same time, I might be saying something which is like, I might not know that time and something. So, there should be someone with the machine, also, from the hospital to verify at the initial stage itself. But, yes, it’s like usually you go to hospital, your initial, like first checkup is, like, blood pressure, height, weight, and everything. So, that is like initial part that’s being done by the robot itself, and the person is like—see’s you. “Okay. Fine. You are done? Okay. You wait here.” And for 10 minute, or maybe in 5 minute everything will be done, step-by-step, and there’s nobody required here. And like, it can be done, so this is how I’m imagining, and then, like, I’m imaging a hospital with several, like, several robots rather than just human beings.

STOP 37:03c

Moderator: Okay.

1: So, yeah.

Moderator: Do you envision—you envision being at a healthcare—

1: Yeah.

Moderator: --I don’t know, an institution.

1: Mmm hmm.

Moderator: We’re you thinking of the same when you described it, or could you—were you thinking you could do it from your home?

2: I was actually thinking of a smart phone.

Moderator: Oh, a smart phone. Okay.

2: Mmm-hmm.

Moderator: How do you guys envision it?

3: To me, I think I would see it as more, like, an assistant of the physician, actual human, ‘cause, like, for example, now, cancer is very common. And there’s so many different types that keeps changing, so I’m thinking it’s a very useful tool for doctors. They could kind of—‘cause then sometimes you just don’t know the most updated information, so for example, the patient describes all these symptoms, and then, like, the virtual physician gets all the data. They kind of like, all understand, and then kinda do an analysis for—the actual doctor, and then maybe from there, like, they can give her more—a broader, like, or more assured diagnosis of that specific disease. So, actually, I think that’s the way more. The human doctor and the virtual work together.

Moderator: So, they complement each other.

3: Yeah.

Moderator: You don’t see—you don’t envision the virtual—

3: But then also, these are for the more extreme case, like—

Moderator: Okay.

3: But then, I think for general, like, normal, like, cold, flu or high-blood pressure, that kind stuff, like, for medicines and how to treat it. These very common diseases, I think virtual physician can handle, but for more high level, like, I feel like is still in combination would be the best.

4: So, basically, that’s what, it depends on the situation, so if I’m having sometime of lower level, I would definitely be thinking of using the machine at my home, maybe a phone or a laptop or something, and I think it would be providing me a drop-down list of shop-listing things, and it could provide me what I’m going through, and it can give me some remedies. And next I’m thinking, if it ask me to provide some test, like, blood reports.

Moderator: Yes. What do you think about that?

4: Yeah. So, most probably it might ask me for entering my numbers, like, what is the reading of my blood reports or those things. Based on that, if it provides me further detail of what action I’m supposed to take, I’ll go for it. If not, I will definitely go to a doctor.

Moderator: Okay.

4: Or maybe what I would do is compare with the thoughts—what a machine would say, and what a doctor would say, and see who’s gonna be right.

Moderator: Yeah. How would that work? I think you raised a really good thing. How would you, like, how can you—you mentioned blood pressure. You mentioned height and weight, so if you go to a doctors’ office, they measure you right there, right.

4: Yes.

Moderator: Now, as you’re describing it, you’re reporting it.

4: Yes.

Moderator: So—

4: So, I would have to still get the check from the hospital, right, and get the readings and put whatever it needs. It’s a little bit confusing. I don’t know what you’re —

Moderator: So, do you also think, like Shelly, that you have to comp—you have to use both?

4: Yes.

Moderator: Is there a benefit to using both?

4: Mmm hmm.

3: I think it should, like, it should be trained—someone trained to give all the data. Like, so if there’s a special physician should be working with whoever taking the blood tests and something—

Moderator: Yeah.

3: --like, it should be handled, like, not you as a middle man, but it should be confidential, and accurate, like, and I don’t believe, like, you can just put—or I want to do a blood test, and then I have these findings [inaudible] I’m gonna tell the virtual that I have these findings.

Moderator: Yeah. Right.

1: So sometimes it might ask in a different terms. Maybe in the report, it is in a different terms. So, what I’m trying to say is, machine has different terms, and my report has a different terms, and I don’t know what it is because I’m a common person. So, how will you put it.

Moderator: Yeah.

1: Right. So, in that case we would definitely have to go to a doctor to validate what is what, and put it, like, all in the machine.

Moderator: So, what if you were to go to a place, and it could be a hospital or it could be a lab, right.

4: Yeah.

1: But I believe, sorry to interrupt, but for this problem, I think we can do scanning, but can, like, I’m sure we are talking something artificial intelligence.

Moderator: Right.

1: So, scanning is—now, it is we can scan a picture and put in google and what it is, it can tell you. And those numbers and this, like, of course, it’s something from the same area we’re talking about. If it’s about the blood pressure, blood counts, or maybe different units, different, but this is all about the blood pressure. And if you are developing something, we’ll definitely try to incorporate as many as possible. So, I think that’s not a big deal. We can scan the data, and then you can upload it, and you can detect out medically. And about the measuring heights and everything, so like I was saying that we have to go, definitely, but I was, like, saying that we can save a time rather than just doing someone, like some human being coming for you, and doing that. We can just have a machine there, you know, and then if you just stand inside like—

Moderator: It sends everything.

1: Yeah. So, this is like technology enhancement.

Moderator: So, there might—you’re envisioning some extra technology.

1: Yeah.

Moderator: It’s not just the physician or the virtual physician.

1: No. Definitely. I don’t think so. It’s just go by—it’s, like, you are shaping your body and increasing just one, like knows, and so you have to balance everything. If you are looking for something artificial intelligence, like, virtual physician, you have to include, like, develop your technology.

Moderator: Okay.

1: So, I think it go hand-by-hand everything.

Moderator: Okay. What about—sometimes you go to a physician, and you describe the problem, and the physician explains it to you, as educating you, right. Well, you have x, y, z, and this is what it means, and this is what it comes from, and this is what you have to do. Can a virtual physician do that too, just as well?

4: Yeah. I think—

2: I don’t think it would be as well.

Moderator: Okay. Why not?

2: ‘Cause a human communication is much different from interacting with a screen, so just, like, consider yourself looking at the Facebook comments of some pages. I don’t know. You would be infuriated. But if people were to just interact with each other on a daily basis, they wouldn’t say those things to each other. From a screen, you are not like that. You are not kind, and so the base—when the media changes, the message changes too. At the end, you wouldn’t be, like, you wouldn’t feel the same. You wouldn’t trust that much that’s for certain. Like, when I see a physician smiling at me, I trust his word more. Like, that’s the way enough for me to trust him more. And even that—that was what changes their reputation, that’s for certain.

4: So, basically, it’s like you’re talking to a person, he’s explaining you everything, so if you have doubts, you can ask him, and he can explain you more in detail. But a machine, it’ll be limited, right. So, maybe it’ll provide a paragraph of order, and you want to ask more and more. It will still limit it to a paragraph, right. So, in case of a person, he will explain to you more in detail. You will get a satisfactory feeling. That’s how I feel

Moderator: Okay.

3: Not sure if something different than—‘cause for my own personal experience a lot of times when I actually go to the doctor, like, they didn’t say anything useful. Like, first of all, I said—I was complain my ankle hurts, but then they wouldn’t help me. Maybe they just never experience this kind of thing, and they just said, “Oh, it’s okay.” Like, but then it’s like it happens once in a while, so I would want to know what happened. But it seems like they that they couldn’t answer my question, so a lot of times go search online to see, and try to analyze myself, like, what’s going on. But, yeah, in that situation, I think maybe, like, the machine would at least give me some idea to begin with. If I say, oh, this hurts, and then maybe they will ask—want to know, at least maybe this option. Like, what about this, and what about that? But if you actually go in the doctor, if you just say, oh, this hurts, they might just, like, try to comfort you, say, “Oh, nothing is wrong.” That frustrate me sometimes when, yeah.

Moderator: So—

1: Yeah. It really depends on the situation. Like, back in my country, it’s completely different. If you go and talk to a doctor, he’ll explain you way more than what you need. Like, too many, so it’s different yeah.

3: Yeah. Actually, I had experience of that, but just, I don’t know. For me, like the West—the West, like—

1: Depends on the person, yeah.

3: --at least to my experience, America, like, they don’t go to in deep. Basically, they kind of—I don’t know. Just my own opinion that unless something actually happens, like, getting bad, getting worse, they kind of just, like, “Oh, it’s okay.” But, yeah, sometimes you just maybe, there some issue that hasn’t popped up yet, but it will eventually happen if you don’t take care of it. But all times they kind of—not really taking too seriously.

Moderator: Taking it seriously.

3: Until it actually, things, like came up. So maybe there’s some preventive action that can be taken, but not all the doctors would tell you that.

2: So, I would agree with more explanation. Yeah. It would definitely provide more, and maybe it’d be even better explanation, maybe, it wouldn’t forget. Some doctors wouldn’t, like, some doctors would forget explaining. “Ah, I was just going to add it and I forgot that.” But it wouldn’t say that. It would explain everything. Maybe you would read everything, too. But for example, if a doctor tells me to exercise, I am more motivated to the exercise. But if a virtual physician does that, it’s like my phone’s telling me, “Okay. You didn’t exercise today.” Ahhh. On your phone.

Moderator: So, you don’t take it as seriously?

2: Yeah. That’s definitely.

Moderator: You don’t take it heart.

2: Yeah. Definitely. We haven’t. Like, if it were to have, like, if it were to write prescriptions, maybe I wouldn’t take them, who knows. Like, it wouldn’t be as serious to me as a real physician. Like, that’s why actually I’m saying, like, a human interaction is more serious.

1: Yeah. So, when everything is, like, finally—listening as we are talking about something, which is very, like, very much related to emotion. It’s, like, about your health, happiness, everything. You do just because you want to be happy, and that it’s emotion, and everything, so yeah, talking to a virtual physician or something, so when you are actually explaining to someone, like, I’m just talking to you right now. And I can see that you are nodding your head, so I feel like, okay, I should keep going. You are agreeing with point, so that motivates me, you know. So, okay. I’m talking something which makes sense to her. Okay. She wants to hear something more, so it’s, like, okay. Yeah. I’m going correct. So, what if it’s a machine, I don’t think so. I’ll be, like, okay. Yeah. You are going good, but I would be like, okay. That’s okay, because it’s encoded to do that, but I won’t find that emotional connection to keep going and saying. So, yeah, sometime when I don’t know what I’m going through, it’s difficult for me to explain to that trouble—

Moderator: So, I hear really conflicting ideas here. So, you’re saying that the—you would be missing the human interaction.

1: Mmm hmm, yup. If it’s like, yeah, it’s like totally—it will depend—so, it’s like, I cannot generalize. It’s, like, it will vary from person to person, so if I’m a kind of very, like, emotional, and I’m scared of doctors. So, it all, like—supposed I’m scared of human being. Some people, like, as a child I was so scared of doctors and hospitals. I would like, oh, I won’t go to a hospital—not the name itself. Like, shaken, somebody, like—so, yeah, but if it’s a machine, I’m like, okay. Nobody will be there; nobody will touch you; nobody will do anything. You just have to go and talk. Okay. I’ll go and talk. I can be, but at the same time, if I need something, like, some interaction from your side to talk about, I’ll be missing that part. So, artificial, like, virtual, this thing is going to take—is going to have a hard time when first reading the trust of people, you know, and understanding. So, it’s not just about creating a virtual physician, but it’s more of understanding, like, a mass, and then, you know, understanding, and accordingly doing the stuff. So, yeah, it’s gonna be 50/50%, but of course, we’ll look for the benefits in time, cost, and everything if can provide, like, yeah. One thing I was thinking is, like, mostly, like, I don’t know – here, at the family doctor concept, right.

Moderator: Right.

1: So, yeah. But in India, back in India, like everybody cannot afford a family doctor. So, like, if, like, we have to go to, like, some particular, like, I have a cold and cough. Okay. Let’s go to this doctor. This is very close, and it’s like, the charge -- appointment fee is way less. Let’s go to that place even though sometime it’s like that. We don’t know whether how good it is, but okay. Like, no, we—like, I’m just talking about the indigent people because we have to look for the cost effective/economic, right? We’re not just doing for the upper-class people, like, for everyone. So, in that case, but if it’s a machine or something, it will be, like, a family doctor. So, like, suppose the app—it’s my responsibility to keep my data because I’m not expecting a machine to keep my data for 15 or 20 years. Like, I’m not expecting, but if I’m, like, yeah, still have my sum of the medical prescriptions of my childhood kept at my home. So, it’s individual’s responsibility for sure, but if it’s a machine, I can say, like, okay, probably I’m visiting, and that particular machine is doing. Last time it had been done by this, this, this, this, but if, like a doctor—no, sorry, you cannot get—meet that person because your risk comes on Thursday. And what if I’m sick on Tuesday?

Moderator: So, the continuity you’re talking about.

1: Yeah. Continuity if, like, if it’s a machine, it’s a good thing.

Moderator: Yeah. Yeah. And yet, Cheryl, you talked about having a real physician, a human physician can kind of dismiss a problem or make light of a problem, but you also described it as sort of reassuring you that everything’s okay. You don’t have to worry. Can a machine do that?

3: At that point, like, I cannot really trust the doctor because, like for example, they said it’s okay, but then, like, but the problem keeps happening. That has to be something to me. Like, you know, like maybe some allergy or unknown allergy or something that’s not very severe, but then—but it’s just maybe this doctor, like, is not aware of it, and then they keep you just hoping that—since nothing serious happened, so it should be fine. But then, I think the virtual physician might be able to give more insight as it has more information. For example, like, certainly I would look up on the internet what’s the possible cause, and they can, just you tell the reason, and what’s the symptoms. So, like, maybe the virtual physician will have more, like, data/experience on that kinda thing, so at least, like it will tell you something that maybe that this is maybe possible then. Maybe you should be more careful with, I don’t know, eating this or that. Then you cannot, like, being more, like comfort. Like, oh, maybe. Even though maybe not exactly true, but at least, like, there’s something that you can kind of help yourself, like. It’s not even, like, medicine, like, helping with like, maybe, all the more—it’s just more diet and in exercise, like, but then at least it shows the symptoms, and oh, maybe you need to exercise more. At least you’re kinda aware. Maybe as other have said that maybe just reading tells you to get exercise. It’s not convincing—

Moderator: Not convenience.

3: --compared to a doctor, but if the doctor didn’t say anything, and then you see something on the internet, maybe at least you’re aware a little bit.

Moderator: Okay.

4: So, for me it’s not convincing.

Moderator: No.

4: Machine won’t convince me. So that’s what I’m trying to say.

Moderator: I see.

4: So, because, when I go to a doctor, so imagine even if you saw me for something for 5 minutes and it was not convincing to me, so I have a chance to go to another doctor and get it cross-checked and have a second opinion about it. But when it is a machine, even if you go to a second machine, it’s the same software, it’s the same result.

Moderator: So, there’s no second opinion.

4: There’s no second opinion.

Moderator: Hmm.

4: And when it comes to a person, you might talk in 5 minutes, 20 minutes, depends, but a machine mostly a paragraph, 1-page of detail, right. How more can it elaborate—

Moderator: And so, it’s only—it’s only one shot.

4: Yeah. So, if I’m not satisfied at the first thing --

Moderator: Okay.

4: So, that’s how it is. I can’t tell it give me some more information. So, that’s what I feel. So, when it comes to second opinion, all those things, a doctor would be much better then.

Moderator: So, could this be a reality? Could this happen?

1: Well, like, I’m not imagining a world where, like, completely virtual physicians. Definitely, like, if like right now we have 100 physicians, like, in a state, I’ll see we can go a 50 by 50. But definitely not, like, more than that.

Moderator: Not replacing.

1: Not replacing. I’m not imagining because this is something where we are doing everything for our self, for our happiness to be healthy, and to, like, to have a good life. Like, I can imagine for other steps and all, you know, but if it’s about my happiness and my family’s happiness—because if they’re not healthy, I’m not happy, right. So, after while you’re doing this which is why I’m doing this because I want to keep growing and be happy and keep my family happy, right. So, this is—health is something. I cannot say that I’ll totally relying on something—machines because being a, like, technical person, you know the technicality, and I’m ready to trust this artificial intelligence because I know the technical stuff. But if it comes to general people, common people, you’re gonna have hard time to make them trust because they don’t know what is the efficiency. But I also, another drawback also because we are the human being. We are making codes inside it [inaudible]. And I’m not gonna trust 100%, and so definitely, I’ll be needing 50%. Like, someone for second opinion, definitely a human being. So, I’m not imagining to replace it completely.

Moderator: But it sounds like you’re imagining it—

1: Yes.

Moderator: --to some degree.

1: Yes. Some degree. Like, rather than just roaming—having like 100 humans here, 50 machines and few humans.

Moderator: Okay.

1: So, that we can distribute human resources to other and replace the brains, yeah.

3: My opinion is that—also, like, if it will just be some degree because imagine, like, it’ll be mostly virtual physician, and then no one want to study doctors, and like, be a doctor, and then, like, what a patient to do more research, and then push it—the medical, like, who’s motivating this ‘cause when you do a doctor, and then you kind of read all these, and then people get motivated, and then they want to heal this specific disease? If you just leave it all to computer, who’s gonna push it to a higher, like, new level? Like, yeah.

Moderator: So, are you saying that it just becomes—a care episode is between you and the machine, and there are no—is that what you mean? That there’s nothing that happens beyond that? Nothing bigger?

3: Yeah. ‘Cause AI, like, it can learn based on the cases, but for example, developing new medicine, new treatments, like, people still need to do that. But if then—I don’t know, if people do not go into the wanting to be a doctor ‘cause everybody is, like, taken care of by, like, the virtual physicians, so it might be just, like, in direct effect with them. That’s what I’m thinking might be. Like, because group, like, how—

1: It will be more toward, like, research something. But like, suppose I’m graduating for a medical, like something, and I want to be general physician, but there’s no need of general physician anymore because we have 50% machines already. So, that will be, like, you know, jobs—but at the same time, I’m imagining, like, then we will have more of research-oriented work on medical. It’s, like, machines are already working. Now, what human being have to do? Keep on going. Like, we already have several, like, material scientists, and then we, like, we have thousands and, like, millions of material scientists but like still not medical and polymer and everywhere.

Moderator: Sure.

1: But still we are working, right. We keep on growing, so now we are more of focusing on, like, industries that definitely [inaudible]. We have given industries making it, so same with, like, hospital. The virtual physicians will do, like, industry, will be similar to hospital. Like, machines, like, the production factories similar to the virtual physicians, and like, how we are doing research, the doctors will be more of doing on, like, any human being, they’ll be doing more research.

Moderator: Yeah.

1: So, I’m thinking it can be very good. You know, we will be more focused on research, and then we can keep on growing, and then knowing, but yeah, and just being optimistic.

Moderator: Yeah.

3: I guess my main point is that the more like the personal interaction, like, doctor they treat, like, people. They have a connection that may be motivator, but more like a motivation/emotion type of thing. But obviously we have—if the machine already knows what to do, then the people can do other things. But also, like, if they’re motivated, as in what drives them to do this job and stuff like that.

Moderator: And you don’t see that as disappearing, so I hear that you’re—the way you envision this is more that it’s a tool, but that the human providers are going to continue?

3: So, to be more efficient, as in like—

Moderator: To help them be more efficient—

3: --like, the doctors maybe, like, they can be—‘cause there’s never enough doctors, so maybe this helping at least.

Moderator: So, that kind of brings us back to the efficiency.

3: Yeah.

2: I think it would start first with the insurance companies, so insurance companies would start these to maybe check how doctor is doing, check how everything is going on. So, I would be a bit more pessimistic about it. Like, just it would be—like, it would start with money as concern. So, if it would continue that way, it would make—allow providers to, like, make, in general, insurance providers to make more money. But at the end, would it provide more health, and at the end, would it provide the public to, like, would it allow the public to trust these systems? And would it enable these systems to be, like, upgraded in a way that it solves peoples’ problems? So, it would be maybe—if I were to assume, like, if I were to be very pessimistic, I would think that maybe these companies would create algorithms that would keep people kind of healthy, so at the end they would need providers; they would need medicine; they would need stuff; they would still need to keep going, but it wouldn’t finish their problems because that wouldn’t be a very good business model. So, I think it would go—because this research and development thing requires a lot of work, and in the medicine area, it requires much more than that. It requires data; it requires politics; and it requires a lot of permissions, so I guess at the end, there has to be some profit.

Moderator: Yeah.

2: And if I, like, from my point of view, it would check the doctors; it would help the doctors in some way, but it would check the doctors, and it would grow from there, I would guess.

3: I just want to add one thing with that. Previously, I said, like, some kind of centralized system, but that also can happen, like, company who develop this virtual physician that’s better, but you have to pay more for the software or something, and then you have, like, a not that good software, then at the end, you’re like, good doctor—

Moderator: Then you have good and bad, anyway.

3: --you always still have something like that, so.

Moderator: Yeah. Very interesting.

3: Yeah.

Moderator: Anything? Anything else about it being a reality?

4: It’ll definitely work if there is a doctor who’s along with it to assist the patients. So, it’s just not the machine.

Moderator: No.

4: There should be a doctor also, to validate everything. Maybe, if not at the initial stages, at the end stages, a doctor should be there to help the patient. And also, the doctor also can know more about the patient than the virtual physci.

Moderator: How do you envision that? Would the physician have to be physically in the room with the patient?

4: That’s tricky.

Moderator: Yeah. I don’t know.

1: I think, yeah. I see it as a journey, like, you know, final realties are like a journey. So, journey in the sense you have to start with putting books together, like, this—like, suppose this hospital is very famous. Some X, Y, Z hospital is very famous. Oh, they got a new machine, and you know, like, it can do that stuff. Okay. Let’s—so, how people will trust that? It should be verified when on, so like, the machine is already, and you just have to be there initially. Like, few years, I would say that journey, we have to struggle to make people believe. Okay. It’s working perfectly fine. See, I’m here, and he’s—because we need someone to verify it, doctor will be there and sitting, and then to verify it. And then, like, the time will—the machine works very great. It did everything for me, and then okay. Like, let’s—and then time will come, and then we don’t need a person, like, sitting over there. Like, we’ll have—like, okay. I’m sitting here, but I will be knowing that someone is there monitoring this monitor. Okay. I will have the trust, so someone have to do that. But I’m not imaging something that just could like, lift, and then okay, nobody’s in the hospital, no human being. Everything will be taken care of by machines. No, I’m not doing that.

Moderator: You don’t see like that.

1: Yeah.

Moderator: Okay.

1: Maybe, maybe, like after 100 years or something, but not at least in my imagination.

3: I think, like, near future, it might not impact countries like America that much, but then it will have a big impact in third-world countries who have no access to the doctors, like, nearby. Then it would be very—it would be a big change.

Moderator: Do you mean that you see it developing there first?

3: Like—

Moderator: Or being applied there first?

3: Not being applied first, but how much it impact. Like—

Moderator: Ah, I see.

3: So, like, here we still have a lot of doctors, and they can ask for assistant, but then for those who don’t have doctors, like, access, then when they will have one there, and then it would have a very big impact.

4: In underdeveloped countries, it would be more helpful.

Moderator: Yeah. Any other final thoughts? No.

1: I couldn’t. I said everything.

Moderator: I really, really thank you.

1: I’m just being very, very positive.

Moderator: Well, that’s—as we said at the beginning, there was no right or wrong here, so it’s very, very fascinating to us to hear your thoughts. So, thank you so much for participating, and I believe that there is a—you’re gonna get an email about a gift card as a small token of our appreciation. Thank you so much.

3: Yeah. Thank you.

1: It was good. We talked something which we never think, like, being, like our research studies, and all a part of that. And—if it’s, like, you are not facing those problems, you don’t even think much about it, right, because fortunately, my family, we have never gone to this big disease or something like that, a day-to-day problem. Like, fortunately, we don’t face much. So, we don’t think, but it’s good when you say and just think about it. Like, okay. What could be the possibilities, so it was good time. Thank you.

Moderator: Great. Okay. Thank you.
