# Supplementary material for: Perceptions of virtual primary care physicians: A focus group study of medical and data science graduate students
Source: PLoS One. 2020 Dec 17;15(12):e0243641. doi: 10.1371/journal.pone.0243641 (PMC7745971; doi:10.1371/journal.pone.0243641)
Supplement: S1 File — (ZIP) [file pone.0243641.s002.zip › Anonymized Transcripts/vPCP Focus Group 4 Anonymized.docx]

vPCP Focus Group

June 24^th^, 2019

Present:

Moderator

2 note-takers

Three 4^th^ year graduate students

Moderator: So, [name] gave you kind of a definition of what we mean here by virtual primary care provider. This would mean communicating with an artificial intelligence system, so not necessarily another person that you’d be communicating with. It could be a voice; it could be a machine, but it’s not a person. Okay. So, let’s just start with what your view of that would be. How would you feel about communicating with a virtual PCP?

2: I would say it really depends, right. I guess because, are we only, like, thinking about online, so not personal? And if it’s—when I only think about online, I would say I don’t—doesn’t really matter to me as long as, like, the system, like, is close enough to a human being, so that I cannot really tell the difference or get frustrated. Like, if you are at, like for example, ask a question, and the system is not able to answer, then there is a problem, right, but otherwise, if the system is able to perform close enough to human being, then as long—‘cause I cannot see him, right, or see whatever. So, I guess I’m fine with that.

3: So, actually, I think of this used for customer service in big companies, if you notice that, I feel like. So, like, for example, a wanna return something from Amazon, so I called up Amazon customer service, and they were asking me—I think it was a—I’m pretty sure they use AI in this thing. So, they were asking me to enter numbers or say some sensitive words, like, the key words. So, based on my answer, they will translate to me, like, connect me to a right person, like a human being for the service. So, with me is okay. Uh-huh. So, actually, save a lot of time, and I guess, money too.

Moderator: So, are you describing where you—the first interaction is the machine.

3: Yeah.

Moderator: And then, based on your response—

3: Uh-huh.

Moderator: I’m understanding that correctly?

3: Yeah. Yeah.

Moderator: Then you get a human?

3: Uh-huh.

Moderator: Okay.

3: Yeah. Mmm hmm, because, like, when ask some questions, the machine may not understand, especially for me, international student, I have accent, and sometimes maybe they cannot catch up. If a human system, help me out, that will be better than machine I would say.

Moderator: So, for you, would you require both, are you saying then? You would want both, or would you be okay with just this virtual?

3: Well, from my side, for my own benefit, if a person could connect me directly, I would be better, but if a company says, if the machine can, you know, use the machine—AI, and also human, together, for them it’s more efficient, I guess. Mmm hmm. Yeah.

Moderator: Okay.

1: I want to say that if just to connect with what she just said, if the AI system, the Virtual PCP, as you said, is just virtual PCP there’s no point, there’s not transmission at any point from the machine to a human, it seems to me that—I know that over time they will try to train the system as much as possible to be close to what a human should do. But I find out that you can’t just get all the issues that could be solved when it comes to customer care or when it comes to patient’s care, different issues will come in different ways that you might not be able to capture, that the Virtual PCP wouldn’t be able to fully capture all the challenges or questions or needs that may be present before our system. So, a virtual PCP becomes limited, if there’s not point of transmission or a common -- or transmitting between the Virtual PCP to a human. So, that’s why you see more intelligent system that becomes more robust, will give a motion when there will be a need for a transmission from the Virtual PCP to the human—to really speak with a human, so I would say in summary, that the Virtual PCP would be limited if there is no point—there’s not point at which there will be—a change, or a transmission from the Virtual AI system as you said to a human.

Moderator: So, it sounds to me, like, all three of you are saying something very similar that you see limitations, and that, if I’m understanding correctly, and that either it would have to be a very advanced system for you to believe in it and to have faith in it, or it would be just a first step, as like an introduction to maybe make healthcare more efficient, and then have a human.

1: I’ve had an experience with companies that depend fully on an AI system, where you know, they just want to really get it, they want to make a point that we have developed our system to just be a virtual system, an AI system where you just have all your needs met, but I find at the end of the day, it’s really not solving customers’ or patients’ needs. Okay? And at the point, it becomes—the patients gets report, like, most of the time they always have it, despite whatever is treated. I just had an experience yesterday. I was looking for an accommodation. I got to this agent where they had—they had this system—totally virtual system, so there was no conversation. At the end of the day, I didn’t get the housing, they lost money because I didn’t get it.

Moderator: Ah.

1: So, my argument will be that, oh, to get a system really perfect—yes, we’re trying to get as close as possible to human, to the human level of intelligence, which is the purpose of AI, or of artificial intelligence. If there is no point at which there will be a switch depending on the need basis from virtual, what from that computer rides a system of having a simulator to answers the needs of whoever is calling to the human to having that customer— being given access to a real agent and real doctor, a real nurse to answer specific questions -- if you’re not being given access, then it’s not really robust.

Moderator: So, if this virtual PCP satisfied your needs, would you still want a human interaction, or could you envision a condition or a situation where, yeah, I’m good with this?

2: I think so, actually. And it’s more, like, based on a case, right. There is case where you, actually, you will always need human interaction, right. There is some doctors you always need to see them, but to be truthful, like, do you really need to see a doctor every time, right? Like, there is at some point where, like a system can actually answer all your response, right, and of course, now, it’s not possible because we just—we have a lot of data, but we just—we don’t have—a lot to be enough, right. There is always a need of more, but there will be, like, that’s like, we will reach that point where we actually have enough data to actually be able to replace the human, which can be replaced. Not everybody, of course, but it’s gonna be, like, a progress, sort of. And at that point, I don’t feel like it would be a problem, right. The human would be able to interact, like, technically with our system just fine. And of course, I don’t see, like any involvement of, like, emotion at that point, which is like something they tell to doctors. Like, keep your emotions, away, right? So, at that point, they’ll be not, like, you’re not really acting as a human, more as a professional. And I feel like machine can do a good job at that, and you can see, like, one example, but it’s not very related, but like, take self-driving cars, right. At this point, you’ll be driving your car, but a human is still needed to be watching what the machine is doing, but I will bet you, like, let’s say 5 years, right, at that point a human, will you really need him? ‘Cause you collecting that data to a point where it can do exactly what the human’s supposed to be doing.

Moderator: So, are you suggesting that with time this could get better and better?

2: Exactly.

1: So, you know, when you talk about artificial intelligence, you know, I would want to agree with him if he’s talking to—if they are moving, the virtual PCP to the level of, you know, being able to operate like a robot, okay. But most times the systems we have when you get to a level, for example, you ask it for something, you call in on the—and the person says, what do you want? Do you want to see a doctor, or do you want to access pharmacy, and all that, or drugs? You have prescriptions? And you say, no. I have abdominal pain. The machine might say, I don’t understand. Could you, you know, it gives a sequence of instructions, like, that it’s programmed to say. But then we have systems that have been developed to the level that it looks like—let me talk about robotics. You know, they have dreamt up some AIs at the robotics level where you have made the system look—to be flexible enough to be able to attend to every questions, every needs of a human. That’s robotics. You know, it’s able to—you want to talk to the system, and then it’s able to, you know, get in, you know, have some cognitive level of getting into the system and give you the right answer, but time. If it doesn’t get to that level, and somewhat of what I’m trying to say that it depends on the level of the development of the Virtual PCP. Some level might not—might still need some form of transmission, okay? But some level, they train the system to actually fully behave like a human being, to be able to think and respond. But how many levels I go, I will want to make certain level of investment. Okay.

Moderator: Well, so you’re talking here, about different levels of communication. Do you see any advantages with this type of interaction in a healthcare? Think now, this would be a physician or provider.

2: Again, it’s very dependent. Like, first of all, for a human—as a human, right, there is some point where you really don’t want to express everything to a human. Like, there is all, like, this condition you—without the fear of like, what does the other one think about you? Like, what is the concept? What are their concept about you, right? But with a machine, most of the time like an ATM, right. No one is ashamed of, like, they’re gonna take $10 right now. No one is going to see me if I have $5 in my account, no one gonna see me, right. And I feel like, in terms of, like, the relationship between doctor and patient, it’s a different sort of relationship, but at the end of the day, it’s like, it’s the same. It’s not really as I’m talking to you has a friend or something like that, so it won’t really matter. Like, that’s like, it’s like that would be the hang of it, I feel like.

3: Yeah. I’ll agree with him, so like, if you have some like a weird feeling, whatever, you can talk to the robot. Like, the machine, and they will give you answer, but yeah. I think it would be good. Mmm hmm. And you don’t have to, like, drop—you know, like, I make appointment with a doctor. I have to wait, and I go. One month is the least, and it takes too much time for me to get there, and I wanna Google. Like, Google my–I describe my feelings, I cannot trust that, you know. Like, so, yeah. So, I cannot trust that. If I take the way, like, for example, Google, like, I type. So, I search, and the Google tell you, you should do this, do this. What if I did and accidentally something happened to me really bad? Who gonna take responsibility, and myself have to take it. So, if any hospital could have provided this kind of service, and also take responsibility what the robot tell you—tell the patient, you know, for that. I think it would be adaptable, uh-huh, ‘cause as a patient, you want advice from a professional person, more like, an institute or whatever, uh-huh.

Moderator: So, what I hear is that it might be that there’s no judgment, right.

3: Mmm hmm.

Moderator: You have kind of a neutral relationship.

2: Yeah.

Moderator: And then it could be more efficient, you said.

3: Mmm hmm.

Moderator: No long waiting times.

3: Mmm hmm.

Moderator: Other?

3: I think it would be cheaper.

Moderator: And cheaper.

2: Yeah. That would be a big factor, I think.

3: Cheaper and faster. I think I really care about, like, for me, a student, I really care about, like, how much I would pay for a visit in a hospital sometime.

2: Yeah. I mean sometime it’s kind of crazy, like, when you meet a doctor for 30 minutes, and then your bill is, like—

3: One thousand dollar, twenty.

2: --like $1,000.

3: That’s how I did. I with the doctor 30 minutes, like, I think less than 1 hour—

Moderator: Did you say 7 minutes?

3: No, no, no. Like, 30 minutes, they asked me my, like, ask what’s going on with my nose ‘cause sometimes bleeding, and then they just check, check, check. I guess, like, less than 5 minutes, honestly, and they take 30 minutes, like half time I need to need to wait then, the process, and the half time they just ask me—record my basic information, and I pay actually $1200. And I was, like, what the?? I was surprised, but I called my insurance right away, and they said the hospital didn’t process—didn’t send the bill to the insurance company, but I was surprised how can be this expensive?

Moderator: And all of that took time for you too.

3: Yeah. Yeah. Exactly.

Moderator: How you could follow up.

3: Uh-huh, uh-huh. Yeah.

2: This is kind of, like, a big issue with where, like, we have doctor over skills for some of the tasks, right.

3: Uh-huh.

2: And then if we can give, at least as a start, those, like, starting jobs to, like, artificial intelligence, that would be a good thing. And I was actually reading, like, following the Google AI, like, for IO, Google IO, for this year for example, like, they actually can prove that, actually, like, an AI system is able to fully identify persons with eye problems better than a human is, right. Now imagine if you have, like, two rooms, like, the doctor is in the Room B, and then you have the AI in Room A, then you can set up just much easier. The human comes in, and you spend, like, 5 minutes. The robot is able to diagnosis the issue faster than the human was, right.

Moderator: Faster and also better?

2: Better. I think eventually we could say that. And then first, we save time. That’s, like, both doctor and I, we save time, and I save money, and then he can have more passion, too, right because he’s able to process more of them, let’s say. Your issue is only, like, maybe, I don’t know, maybe your vision is a little bit off or something. There is no need for you—for him to come and spend, I don’t know, 30 minutes just to looking at you, try to figure out what’s going on, while the AI could do it in 2 seconds just because they can—they have more bandwidth of, like, can see what’s going on, like, in a broader than a human can. A human have to pinpoints, right, while if I launch, like, a camera at this wall, and I’m able to see, like pixel by pixel what’s going on, and yeah.

3: Yeah. I agree, and also, like, you know, like—I know, like, some—you have some, like, some certain hospital, they recall your information in your card, so you just need to scan the system with everything. So, if we can’t advance more—if the AI machine use this thing, they can make a, like, you know, read the information in second and know what’s going on with you. Like, if other doctor they use, like, they pull all the information about a patient, they’ll probably gonna spend at least 30 minutes if you want, like what’s really going on. And the one more thing is, for example. We have a really good doctor around the world, in different countries, I mean, yeah. So, if use AI to, like, learn from them, and you don’t have to go to that country, go to that hospital, and make appointment, and get a treatment from them. Uh-huh. So, yeah, if we could develop this, we can—every patient could use good doctors, I would say. Uh-huh.

Moderator: So, you get—more people get exposed to good skill, good competence.

3: Yeah.

Moderator: Through AI.

3: Mmm hmm. Like, a—I’m from China, so if I go to hospital, I wanna make appointment with a really good, like, an old professional doctor, I need to pay extra. Uh-huh. Otherwise, I pay less to the new, you know, like, the interns or whatever. I don’t think it’s intern, but like a—

Moderator: The less experienced.

3: Yeah. Yeah. Yeah. Uh-huh. So, well, you know, you gave these skills to the robot, why would I, you know, it should be all equal. Uh-huh.

2: Mmm.

3: Yeah.

1: So, I want to say that still the same ground, you’re asking for advantages --

Moderator: Yes.

1: Yeah. The advantages are both and also there disadvantages is the development of all of them. Even come into medical care, the different levels of variation, when you talk about the needs of patients. When I talk about doctor visits, seeing the doctor, or having to discuss with the doctor, okay, some doctor visit consultation. You know, every case is different. In as much as you want to use AI, I’m definitely in support of AI, but AI—I agree, one of the point he made. He said some level you use AI, and some level you should use—at some level you should use AI, and then on some level you should use, I think, you should see the professionals. I’m not totally agreeing with depending totally on the AI because someone’s headache is different from another person’s headache. They are quite different, you know. There are different ways that someone who had a case of hepatitis, the issues, the symptoms, the experiences are different. Okay. In as much as, yes, we may need to invest so much and dream up a system, okay, having an AI system. I think it’ll be fine if we give some section of healthcare to AI, and then some window—for an interface with a real human. It makes it a more perfect system. Now, there’s a psychology with patients seeing doctors. I know there’s a psychology when patients are away, that this is a virtual system. Okay. They might develop a feeling of satisfaction, so to have a perfect system, you need to define. Let’s say, talk about, okay, when the patients need to see a doctor and have some discussions, there is this feeling of satisfaction when I talk to a human. Okay. I’m a person. Okay. Have some level as a counseling, the doctor provide some level of counseling as much as he’s providing, he’s trying to diagnosis what the issue is. And then you get to the laboratory section when AI can be fully involved, they diagnosis the section of virtual healthcare.

Moderator: So, you’re saying that part could be AI.

1: Yeah. It could be AI.

Moderator: AI.

1: And then, for example, having to scan, they’re going to give that to – to have robot scanning. And when a doctor sees a patient, you might have to—you go to the same place and all that. For some such level of evaluation can be given to an AI, the AI can be evolved, but there are times, you know, in healthcare we have primary healthcare needs, then we’ll have secondary. We have times when the issue becomes really—you may need to see professionals maybe, consultants. Okay. Somebody who is a specialist. Okay. At the preliminary level, the basic level of, okay, of I’m entering my patient, you could use AI. But when it gets at some level, there should be some form of connection with—have an interface with a human.

Moderator: So, it sounds to me like you all hear advantages, but they’re not enough, alone. Those advantages are not enough to convince you that this could work.

2: I agree with you, like, in one sense for, like, you—totally agree with you, we will always need some human interaction, but I feel like we—it’s really been on the case, right. Like, I don’t really see—I have appendicitis, like, for example, why would I see a doctor, right? Like I know what the issue is, and then, like, a robot is just—if the robot is just good enough do the same job as a doctor, why do I need to see him? He can do it better, and it’s more better, faster, cheaper, right. So, again, like that interactional, be like to depend on cases. And then also, like, when it comes to diagnosing diseases—

1: If I could interrupt for a second - it’s just to ask a question. Before it got to the level of saying, okay, you have appendicitis, do you think you need an interaction with—before I get to the level of being confirmed, I say, okay. Let him go for a while, appendicitis test.

2: Yeah. Yeah. Okay. So, that point I was going into the second point, actually, because let’s say there is a disease, like, let’s say, not a disease, but a symptom, like, a headache, right. A doctor—well, how do a doctor determine if you have some diseases? They will look at your file, right. Pick up, like, your past, like, medicine you are taken recently or like, way long ago, like, your family history, right?

Moderator: Right.

2: And, like, I think that—like, if we have an AI or it would do the same task. He would look at your file, right now, find your past, and then put it together, and I feel like because AI just way better at looking at data, right. It’s not replacing a human, but like, one thing, I don’t wanna say, like, they’re replacing us as a human, but they’re just making our tasks efficient, right. So, something we could do. Maybe I’m sleepy, right. I could like mess up something, but I’m pretty sure if, like, you have an AI you can also have redundancy, or you can have the one checking over the other, like, way faster than human can do to do it right. And at that point, I feel like the robot or the AI will be better diagnosing human, and that’s how the very good for example at capturing cancerous cells, right. Like currently, well actually I’m not sure how it’s currently done, but last time I checked, so you would have human actually looking at that—those samples of cells and try to determine which one are sick are not, right. And AI can also do the same thing, and then from that he can go on and say, okay. Maybe you need to do this, whatever. And—

1: Now there’s some level of AI that Google does right now.

2: Yeah.

1: Even for—where you getting the facts. Let me give you a real situation. Yesterday -

Moderator: Sorry, do you mean for health or do you mean AI for health or?

2: Not really health. In terms of you could check in what is wrong with me. Okay. I have headache. Okay.

Moderator: Oh, you mean like that. Okay. That sounds a little bit where you were getting in at. You Google it.

1: Yeah. Google it, yeah. And when we—same computer, same system, same AI system. They have a [inaudible]. You get in, you have headache, it will give you all sorts of suggestions, which are correct. But the situation on the ground could be different depending on the human, depending on the experience. For example, I had some feeling of numbness in my hand. I could not—when I woke up in the morning, I could not really hold things. Okay. But after some time, it gets better, as of the day. Now, when I put that into the system and ask, let’s say it’s a virtual system, right now. The virtual system would think of diabetes. The virtual system would think of the nerve damage. The virtual would think of all sorts of things. You get it?

Moderator: Yeah.

1: So, it might be endangering the patients if—depending on what AI has been developed, but if there’s some form of, some level of, okay, a physician, you know, having an input, it becomes the perfect tool—

Moderator: Well, so remember that. Now we haven’t really defined it, but when we say a virtual PCP, a primary care physician, then we mean that this machine has input, right.

1: Yeah.

Moderator: So, it’s not just the whole cyber world or internet, which is more of what you might be getting when you google symptoms, right. So, here, it’s a little bit more refined, but I see—I think we’re—that leads me kind of to my next question is what you see as drawbacks. You definitely saw some advantages. What are some of the drawbacks? Maybe what you’re describing would be a drawback that you get bombarded with possibilities, and that you don’t know what to do, what to do next.

1: Yeah. One of the drawbacks could be information is given to the patient without some form of helping that patient to digest information. For example, it tells somebody you have hepatitis, and the person is depressed and leaves the hospital in a very dangerous state.

Moderator: Couldn’t that happen—couldn’t that happen with a human doctor?

1: With a human, I think doctors are well trained to have a better way of communicating information in such a way that, you know, yes, you communicated with the patient, but not the patient will pick it up and still be calm to a certain situation. Then you say, okay. You have hepatitis, and then the patient loses control and becomes—you get the issue. You’re -- okay. But the AI could be—there’s a lot, again, to after having presented information, I think we’re gonna think about—having a way of communicating information and then giving the proper counseling and calming the patient. It’s possible. Okay. So, yes, we’re gonna save time; we’ve gonna save money; we’re going to have doctor do some other specialized task. Okay. But then it’ll be fine once we are mindful of some of the drawbacks, some of these gaps. You can have that built in the system. Okay. To ensure that we get exact same value that we get when we see a human.

Moderator: Okay. Other?

2: I think I have two. Like, one of them after I thought about it when 3 was speaking, is who gonna take responsibility of what the AI is doing? That’s one thing I saw, and then the other one is, like, part of the implement of AI. Like, this is kind of the—it’s kind of an ethical question I think in the AI world, and especially, like, for example in the deep learning subset of machine learning, for example. Where let’s say you train a machine with biases, right. Like, you can have genetic biases or some sort of, like, social biases, and so, like, when we’re implementing those AI, we have to make sure that we do our best not to put in our human biases into the system. And I feel that’s probably the only two issues, I think.

Moderator: Okay.

3: Yeah. For me, I feel emotion support. I don’t think AI would do such thing as humans, say how are you? How you feel? Like, some words make the patient feel comfortable, uh-huh. Like, I don’t know, for me I—I’m kind of, like, a millenial I would say. I like to—I accept new things, uh-huh, and I’m okay with AI to, like, check my body, whatever, but for the people, like, my parents, they would be a little be conservative. Like, you know, when you, like, you come into these machines, you go check by machine, it’s just like—what’s that feeling that’s cold? Just you feel cold. Uh-huh. A little bit.

Moderator: It’s impersonal.

3: Mmm hmm. Yeah. That’s how I feel. Yup.

Moderator: Other? Other drawbacks or disadvantages?

2: Maybe trust-ability. Like, how do you tell that what the robot just tells you is right, right?

1: Exactly.

2: But again, I feel that this—this is something that’s gonna be set up here with time. Like, it’s just of data again. Like, the more that you have, the more accurate you get, right. And it’s also, like, a model of, like, desensitizing people. Like, right now for example, we were at this conference. I just talk, actually, at the conference, but about, like, AI in actually, like, automation of, like, let’s say cars and whatever. People actually say, like, oh well, like, you can just disturb an image on the road, and it will think there is a human or something, and then, right? But then, I can’t start, there are also researchers working on how can AI detect, like, a false data or something, like, that. So, it’s just a matter of time. Like, we just need to tell people that, okay, don’t be that afraid of it. Like, it’s just, it’s a process. It’s getting better and better. It’s not perfect, and for some reason I feel like human are not perfect, too. Like, not all doctors are actually good doctors. So, yeah.

3: I kind of agree ‘cause last time I went to see doctor, and I was kind of a little bit anxious, and she just tell me, “Do you want some medicine?” And I was like, why would I need medicine? I mean I just little bit nervous, anxiety. Kind of nervous, yeah. I don’t need a medicine to, like, calm me down. Uh-huh. I think this is wrong, like, I don’t know. It’s because, like, you can make more money for the hospital or something. I don’t think it’s right. Uh-huh, and if we use AI, fortunately, this is not gonna be a problem. Like, they wouldn’t just give you medicine because you’re nervous or whatever. Uh-huh.

1: The only one who would do it is humans. Sometimes there are levels of, yeah, issues that you see the physicians, the doctors seek a second opinion.

Moderator: Yes.

1: They come to check each other. Okay. So, yes, for human, yet at some level they check themselves.

2: Yeah.

1: As a patient, you’re also free to get a second opinion if you are not satisfied. So, I’m actually—we’re not right, it’s possible it’s happening. I think it’s happening right now, but I’m not experienced as a person. Depending on the AI, okay, system you may also want to say do I really believe what—just what he said—what the system just told me. You may want to also seek some form of… So, I think it comes—it will gradually move when people are certain, so the system, possibly after seeing the fruits, the performance over time. And as he said, over time the system will be improved upon, and then—

Moderator: What if you try a meeting with a virtual PCP and you’re very satisfied? Do you think then you would go back?

3: Oh, yeah. Mmm hmm.

M: Yeah.

Moderator: You would, you know, would you trust it? Maybe what you’re describing is that, you know, the more—the more that I think you both—you’ve all three of you’ve said this. That the longer it exists, the better it’s gonna get. Maybe that’s the nature of AI—that’s the nature of it. But also, what about that idea of feeling that, yeah, I can rely on this?

1: That’s the ability the doctors have is that every patient is different. You can be fine with headache. We are different. No one will respond to my headache—it’s different.

Moderator: So, you don’t think that a virtual PCP could pick up on that?

1: No. The difference?

Moderator: Yeah.

1: Okay. If we now have AI system that has already analyzed the situation, the DNA information, you know, DNA identity can [slaps hands] data.

2: I think with sufficient data, with AI, it could me more—it could be good, yeah.

3: I think it really depends on time. So, I would try small steps first. I wouldn’t try the big steps. I wanna, you know, I wouldn’t go by myself. I would see how people try it. How it, you know, like, how it works. If other people say, mmm, it’s really good. I got cured, and I like it. Then, okay, I will try. And then, you know, from small to big. I even truly accept it, you know. Uh-huh. It’s about time, and also about word of mouth, I guess. Yeah. Uh-huh.

Moderator: You think maybe you would gain acceptance over time.

3: Mmm hmm. Mmm hmm.

Moderator: Depending on your own experience, and also, maybe depending on what other people said.

3: Uh-huh, uh-huh. And also depend the treatment. From small to big, you know. I would trust small first, small treatment first, and I would increase gradually. Uh-huh, and then I build trust on this machine. So, I would just go for it if next time I have a problem.

1: As a person, I’m really, if I were to have kids, I wouldn’t advocate for a system that is totally dependent on AI. What happens to our doctors? What happens to the particularity I would get from, you know, that we see in our patients every day in the clinics. Okay. So, yes, wonderful. Excellent. It’s gonna be efficient in some stages of really getting to finish all the task of satisfying the needs of the patient. Okay. From the clinic, down to the labs, okay, down to pharmacy. In fact, the AI would do very well in pharmacy. It would do very well in the labs. Okay. But AI would do well, very well in some form of other diagnostic evaluation. Scanning and all that, but when it comes to that portion that we normally present for the doctor to have his formal to check and really check the particular difference of what the patient—what the patient is presenting. Could it be different headache? Could it be genetically informed? Could it be some experience at home that is different from Patient A that came with same headache, but you know—

Moderator: And you don’t think that a machine could do that?

1: The machine could do that, but to a level, to a limit. Okay. Do it to a level, and also, you’ve got a provide this—this is one of our systems could also be a trend. You know, it’s good for all of us to understand what this AI is. You know, over time, we train the system like he just mentioned you know, network, and all that. You have to train data, okay. Train the data to work safe. At a point you evaluate that data whether it’s really correct or not. Is it saying what’s it’s supposed to be? If it’s some small percentage of data. If it gets 40% of that dataset, information that you give, is it going to send the same thing as when it gives 60% data? Okay. You find out if that system is accurate or not. That is what the big deal is all about, and then you do, you have all the models. Neural network, official neural network. Different algorithm just to check to make sure there’s a particular – you pick a particular need to want to solve. But you use different models to find out if what you have said, using a particular model, is true when you use another one. Okay. So, all they are doing AI, just train up the system to behave like a human. But I want to say that humans are also unique. The way they present today could be different from the way a human will present tomorrow, but over time for many years, for over—when you’re doing the sample together, and you organize over time. You could be able to capture, to train that system to know how a human behaves. Okay. With sufficient data. That’s what --

Moderator: This is all something that would develop. You come back I think many times to this idea that this could develop. This could become more advanced and maybe better.

1: Not if – what if an event that has never been present, that was never used to train that AI system presents itself in the future, what happens? That is a limitation of AI.

Moderator: Good question.

2: I feel like, for me, like, I really feel like at the end of this our sense of fear, like, as human being, like, in a situation if you go couple centuries ago when they’re introducing some medicine, like, for some diseases, human had fear or they were reluctant toward it. But as, they actually see the results, you know, like, then they become more friendly toward it, and I think it’s the same thing with AI. Like, right now people are fear, like, some of them is beyond the concept of they’re gonna take our place, so they’re gonna take our jobs, and that’s one thing that’s—I don’t wanna say it’s true, but it’s my feeling—I feel like for some doctors that they are afraid of that, so they will rather than say, okay. The AI will do the job worse than we do. But at the end of the, like, maybe if they can understand that the AI will be there to make their job more efficient, then maybe AI should help them to do their job, then maybe they will sensiblize people and tell them, hey. This is actually your friend. This is not your enemy.

Moderator: Yeah. So, how do you envision—how would it actually work? If you could describe how you think it would be to consult with a virtual PCP? Like, you talked about—I thought it was really interesting—almost like you bring your card and you stick it in the machine, and then they know everything about you, right.

3: Uh-huh.

Moderator: Like a chip.

3: Mmm hmm.

Moderator: Is that the way you see it? Is that what it would take, or do you see it as something that you do from a computer, wherever you are, or do you have to go to a specific place? I mean how would you envision this working in society.

3: Yeah. I would say, how ‘bout if you have all the records and maybe you don’t need to go to hospital. Like, you know, you could put a machine in the supermarket, and if you don’t feel well, you type what’s going on, and you slip the card—all the information on that show up in the machine under the MMIs. And in order to do this, like, in order make it better or accurate result, you may can do something [inaudible]. Like, put all the information about yourself, information in the machine in the chip. So, yeah, you can just, like, off at the grocery store and you can just check what’s going on with yourself. Uh-huh, yeah.

Moderator: What if the machine says to you, okay. You need to check your iron levels, and your Vitamin D, and your HbA1C?

3: I think you can check it from blood test.

Moderator: Mmm hmm.

3: So, probably if the machine has something, you know, like, you just put your finger, they clean your finger, and get a drop of blood. And, like, 10 minutes or 5 minutes they’ll analyze the result.

Moderator: So, right there.

3: Uh-huh, and they say, oh, okay. What do you need, like, for example, you can change your habits. Your food may be heavy or whatever, and you got information. And, like, 7 minutes I think maximum or something. Uh-huh. I think this is the future. Like, how human beings should live. You think, like, I would think, like, 60 years ago, back my, like, in China, there was so many people don’t even have food to eat, uh-huh. That was a really bad time. Like, people—some people even in order to survive, they eat the tree. You know, the skin.

Moderator: The bark.

3: Yeah. Uh-huh, to survive, and then now, just only, like, 60 years after, you look back to my country how it looks like. So, how ‘bout from this period, the future, farther period, I think, you know, should be more developed, uh-huh. And the health is a major issue, and there should be, like, a path—there should be, like, a timesaving, and money saving, and also convenient, I would say. Mmm hmm. Mmm hmm. You can put that machine in the company, the headquarter, whatever, and just check if you feel dizzy or something. Uh-huh. I think.

1: There actually are no limits to development and technology. After I’m thinking I will be speaking very well, the level as it comes of the system, you just presented, maybe by level of—possibly depending on my level, my interaction. Now, I don’t wanna sit and be imagining, okay.

Moderator: No, but that’s what we want you to do. How would you imagine it? That’s exactly what we wanna know.

1: Yeah. I’m just trying to imagine it, but for example—

Moderator: There’s no limit. Whatever comes into your head is good.

1: For example, you fell, and you had something somehow behind you, your wrist, your hand. Okay. I imagine, okay. Normally, natural—what I’m used to, I’ll get results from the doctor will say, “Okay. Remove your sleeve -- have some observation done.

Moderator: Okay.

M: Okay. And the human will be very flexible to check you and will get to see the situation of that injury. Okay. And the level—how severe the injury or the nature of that injury which cannot be defined, nature. It depend on what he will do, the doctor will do, so I’m also thinking this AI system, is it able to have—observe me and tell me to turn my wrist, my hand, and all that, and have the observation done, and then, okay. This is different. This is—you are told you had—you’re thinking that they have not done it, but no. You don’t get that level, okay. A machine would prescribe this. I’m mostly imagining that. Is it possible?

2: I thinking more something that’s go from portable device to full-blown size of let’s say the place people try their dress on, right. Like, reflect my watch, for example, now is monitoring my heartbeat, and then when something goes wrong, they tell me, hey. Maybe you could check your heart right now, right. And at that point, I would then go to a place, like, a supermarket where they a closet-sized thing I will go in, and then it will scan, like, a round scanner

3: Uh-huh, uh-huh.

Moderator: That’s very interesting.

M: And then ask me some questions, and—

Moderator: You go to a central place.

3: Yeah. Mmm hmm.

2: Yeah.

Moderator: Very interesting.

3: Actually, easy. When you go to airport, they scan you with a machine. You can adapt in the health machine, whatever, like that. And also, for your point, I will say, you don’t really trust AI because you think it’s not a fully developed, but I think first of all, depends the time. Second, when you say you prefer doctor, for example, say, if you prefer doctor to check your body, like, examine your disease, they actually doing a other artificial intelligence. They just do intelligent. If they do analyze, use their brain, and you know, like a human brain are not really fully used, the most of them. Uh-huh. They cannot memorize everything. Like, you know, like for example your 20 years—being a doctor 20 years, and you have a really good experience, but you cannot remember every single patient. How they—what do they look like, and like, what’s the disease look like, whatever. They may make mistakes. Uh-huh. And by using AI, I would say the AI can record the human information, they could actually do some analyze, like, I from Asia. My body is different than European, like, American, like a this, you know. Mmm hmm. And based on this kind of gene, they can analyze a better solution for you ‘cause for example, you look, you take a doctor from here, and most of patients they check is from here, local here. Uh-huh. So, sometimes they’ll have to define, like, what a real treatment for you, uh-huh, you from different region. You drink different water. You eat different food, uh-huh, yeah.

1: So, are you saying that based on this information, AI is better—

3: Yes. ‘Cause AI can record all the information—

1: --it captures the differences in race?

3: Mmm hmm.

1: The difference in race, experience, and all that?

3: Yes.

Moderator: I think that what you’re saying is that the machine might know something that an individual physician—

1: Would not know.

Moderator: --might not know if that has not been his or her—

2: For example, they can take into account, you can connect all those computers, and then they become a single one, almost like, you need go to the point of, like, double checking themselves. Let’s say this one doesn’t know, it cannot treat A. Computer 001, like, have you ever seen someone with this and this and this? And they say that computer, oh, yes. This one had a patient and took every symptom, and this what the issue they have, and then, right, that this can be done in [snaps fingers] a minute versus when you have doctors. I’m pretty sure doctors do that too, like, contact each other and maybe say, have you seen this? But it seems like a day or something like that, I would say, maybe.

3: Oh, yeah. Mmm hmm.

1: But this comes to what I’m saying. He has an experience. You know, he has gone in—send someone going to a system, scanned and all that. Okay. That’s comes to—I wanna make a statement based on that. If we publish, okay, and say that AI is better, okay, in terms of patients and all of that, what it should be included that it depends on type of AI and the version of AI. Another hospital A, or Z ‘cause say we have walk-in. Okay. AI is acceptable and they are using AI, but the form of AI they are using might not get to that level of complexity, that level of—to really take in of all patient needs, so it depends on what AI you are using. You can see a level of understanding of AI is different.

Moderator: Yes.

1: And also, the level of AI that different hospitals use, different systems we use are also different. So, what—

Moderator: So, is that so different from what we have—from the reality we have with human physicians? What the physicians at Butterworth, here, up the street, are those physicians the same as the physicians down the street at Mercy Health St. Mary’s? Are they the same? How do we compare? Are the systems the same? Are they basically the same or do they have inherent differences? As patients, how do we know?

1: When you talk about AI, you talk about the computer world, okay.

Moderator: Yeah.

M: Then version comes into play. Version or level of complexity or level of sophistication. Maybe the word sophistication comes into play. Okay. But when we talk about human beings in Michigan, in the US—

Moderator: In Grand Rapids.

1: --in Grand Rapids, there’s a protocol that hospital healthcare depends on, which you can say is the standard of care. Okay. There’s something called standard of care, so we depend on standard of care to say, oh, yes. We are, yes, doctors are different, but because of the standard of care, I have the confidence that I’ll get good quality of care.

Moderator: Well, that really brings me to my last question, and that is whether this could be a reality? So, what you just said, a standardization, would that be possible with a virtual PCPs, also? Could there be an accreditation bureau or something? I mean you’re right, of course, that there would be different systems, but how do you—you know, you all had such good ideas about how you envision this, could it be a reality? Now, we’re in 2019, could this be a reality in 5 years, 10 years?

2: I thought by asking a question to all of us, let’s say, like, how do you make sure that we all kind of like follow the same rules in this room, like, you can’t really tell, right. Because you don’t know what I’m thinking. You don’t know what anyone of us is thinking, but if you, actually, like, unit in this room, like, profession would say, I will take into an example that’s said how to define, like, a network. How do you say the standard for, like, network? So, we have a group of like professional, like, a very, like, proficient in the field, and they will talk together, and then they will define what’s a minimum requirement.

Moderator: Standards.

2: Yeah.

Moderator: They write the standards.

2: And that’s it. You just much easier to do it standard of an AI than human beings, honestly. I mean this is just what I feel like.

Moderator: Okay. So, do you see this as being a reality? Could this happen?

2: I think it will happen.

Moderator: You think it will happen.

2: Yeah.

3: It’s just like, you know, like—I mean, like a 10 years ago when you wanna make a new key, like, make a key, like copy a key, right, you have to go to store. Now you can just go to Meijer, same thing. Uh-huh. Like, maybe just bring a laptop and make minimal it, like make is smaller, and then bring to somewhere, uh-huh, and make, like, a much faster. Mmm hmm. And also, like, for example, you see doctors take—you had your headache, and they have to go so many machine, like, a check of what’s going on, what’s going on. If this not working, then check other—couple machines, see if this work. Yeah. But if you use AI, they could, like, have a better decision. Like, they make the decision would be faster, and that they—you don’t have to go through each department to check which kind of, like, for example, you need to do blood test, pressures, whatever. You can just do at one—

Moderator: So, you envision this as, like, an all in one.

3: Yeah. All in one, uh-huh.

Moderator: Okay. You do it one-stop—one-stop shopping I was gonna say.

3: Uh-huh.

Moderator: But you do it all at one visit.

3: Uh-huh. Yeah.

M: I think that’s possible, too, like, from your idea because you can put multiple knowledge into one AI, and you can put just—a human cannot be [laughs] a pro in everything.

3: Uh-huh. Yeah.

2: Everybody have their own field, and it’s a good thing for us, like, I should, you know, make us different in a certain way, but as professional, like, in a professional manner you rather have someone, like a knowledge base, right. Like, something that has all the knowledge that everybody can draw from. I think with AI, like, something like that.

3: Mmm hmm. And also, like, when you go see these scan, they have a big picture, and the doc always, like, a—see the pictures through a light to check on what’s going on with your bones. But if you use AI, the machine would go automatically check which points goes wrong, and you know, much clearer and much faster, right. Uh-huh. You just, like, avoid this step for the—avoid this step to check the pictures.

Moderator: And it sounds like you think that would even be a better—you’d get a better diagnosis.

3: Yes.

Moderator: Plus, faster.

3: Uh-huh, uh-huh.

Moderator: More accurate.

3: Yeah. Sometimes the shadow—I watch some TV show, like, you know, the shadow of people—don’t—really—can’t see well with human eyes, but machine do well. Mmm hmm. I trust—

Moderator: The machine would pick up the nuances.

3: Yeah. Mmm hmm. Just need to get a good data and analyze that all again. Mmm hmm.

1: And I think is better—these are, like, experienced, okay. Like, just to speak because also remember. I mean suppose AI, but I’m also thinking—I’m trying to imagine all these. We have different systems in the lab as we speak, and sometimes we do what we call the mistake test of evaluation for some of these systems to see, okay. What is—you check three systems together, and three systems diagnosing just one disease, and you find out that these three systems don’t get it accurate. Okay. They don’t get it accurate. The level of accurate is not 100%. This one get it more accurate. The number of positivity of a particular system maybe higher than the other one, and the number of—let’s say they call it specificity. We begin to differentiate those who are not—those who don’t really have the disease from those who have the disease. Okay. It’s higher specificity, for a particular system than the other, and then you put all these computers together to get your real accuracy. So, I want to say, yes, it’s fine to have a system, but it’s also—what I would recommend is having a system where some of those tests are also rechecked by—double checked by a counter AI system. Okay. Just, like, we humans do all—just as we humans also evaluate the methods of tests. I don’t know how it’s gonna play out, but—

Moderator: But that’s how you envision it.

1: Yeah. It would be better for us to just get into a system at the very added time of, okay, those enjoined – Getting into a system and then says, okay. You have this, you go for this. And if you check, it might not be accurate because AI depends on what information you make it validate. Okay. Like, she brought the example of the scan, the shadow and all that. Depending on the lights available, the image available to the AI it could take a position where we find out that these scan systems don’t make—don’t get exact results. Don’t make 100%. A statistician might be able to experience that. If you have done different studies to really find out that there are some mistakes that the systems also. I just hope—I wish that the AI system it captures all its gaps and have a better system.

2: I think I agree with you, but I still feel like that it’s really a matter of, like, the implementers. Like, adjusting to like—they have to be ethical enough to actually think about, like, issue like, system redundancy. Where you have instead of just only one computer, maybe you have, like, maybe five or six or something like that. So, ‘cause at the end of the day, if you have only people, like, a hospital trying only to cut the costs, then they will ignore, like, there’d be like—they won’t take into account the patients. They will be like leftover, right. But if, like, the implementers in the hospital are responsible enough to actually invest enough resource into, like the system, I feel like we could actually have a system that is viable. And in my opinion, like, when those systems would be available, like, when they would come out, they will be at the same level or better than human. And as time goes by, they will only get better. And the reason I feel like they will be at same level or better is because AI only do what’s, like, we have done in the past, and we’re doing now. And that’s how they’re being trained, actually, ‘cause you train them on the data that is past, right, and they adapt on that. So, they know all our pasts, and then from this moment x, they will only learn more and more, which would mean they will actually learn faster and because also—and again, the issue of, like, they can connect to each other. Like, an AI here can connect to an AI in China, and an AI somewhere in Africa, in South Africa, or somewhere in Europe. Again, they leverage more data, and the more that you have, a better doctor you are. So, I view it that way.

1: Which is beautiful. But also, when you said they get better; I’m also looking at the cost of getting where everything gets better.

2: Mmm hmm. Yeah.

1: I’m considering the number of deaths, okay. So, it’s gonna be number of deaths is going to be, you know, we have—

Moderator: You mean what’s gonna drive this?

1: Yeah. What’s gonna drive that, okay. So, as it gets better, I still hope that a human in some where checking that, okay, the system is accurate, is 100% accurate, so we can go or let’s really flag this issue. This is not a good decision, okay, until it gets better. Not allowing the AI to take decisions, patients go for certain surgeries, or go for certain medication, and die over time from the number of people dying until we get to a point where we just have two people dying from 100. We’ll say, yes, it’s getting better.

2: I think that’s true, but—

1: And because I see, if you look at the world, everybody, you know, people are ambitious. Those who created this AI systems want this AI system to go into the market. Okay. Let’s not just be dependent on that. Let’s have a system. Let’s have a system to check and be sure that, yes, it’s really fine. It’s really convenient, and it’s safe.

2: Again, you’re totally right, again, but I feel like we always have this, like, what they call for example in engineering, control engineers, right. So, like, when the machine is doing the task in their workshop, and then you have this human checking, like, you know, like was this product right or wrong. Of course, assume we gonna totally have that, and again, toward the issue of ethic, then we have to make sure that those company which are like implementing those systems, we just have to make sure that they’re not implementing a product just for the sake of having it tomorrow in the market. So, again, that’s where we need, like, a standardization committee where we gonna bring, like, some professional in the field, and develop something what they will call, like a minimum product, and everybody who trying to implement this sort of AI would have to meet those requirements before they can actually send it to hospitals.

1: Exactly.

Moderator: So, that was kind of like I said, like an accreditation—

2: Yes. Mmm hmm.

Moderator: --organization. You need a set of standards, a set of requirements—

2: Yes.

Moderator: --to follow as a guide. Our time is pretty much up. Any other final thoughts or—

1: I wish I could ask you this question. I know you have been objective as a researcher, but I wish I could read your mind. Because you just said I like the AI, but it’s good to also define what it will be. When we’ll have this beautiful system taking care—okay, we have very efficient and very accurate system taking care of people—what will be the role of the humans?

2: Yeah.

1: I would like that question to be answered.

Moderator: I must—I think you really—you discussed this very well with a lot—really a lot of thought and a lot of nuance to this. It’s not—I heard from all of you that this is not just black or white. You know, that there’s—there are maybe fears with this as well as possibilities.

3: Yeah. I just cannot—I maybe not a really professionally in medical field, so other doctors actually, kind of like AIs adopting different area, I guess. Like, you know, like a scan CT machine of, like, the—some, like, a check your diabetes, whatever. I think they use a little bit of AI, uh-huh. Not much, but the human just, like, a doctor make decisions, they just make decisions. Like, the best result from the machine, and then they make a decision. Now, like, take this task to the machine to make a decision I think, you know.

1: Maybe AI will be preforming surgeries and all that.

Moderator: Yeah.

3: What you said now, yeah, the AI perform surgeries. I heard, like, the doctors stay at home or stay somewhere and the—maybe one hospital, and through the internet to other country, they use the robot to do machine to others. I think this is—

Moderator: It’s starting to happen, and it’s also in the future. Yeah.

2: So, don’t we know what’s going on, like, in the field, actually, like, ‘cause I know you guys are doing your research over, like, how close are we exactly? And to be more specific, what do doctors think about it?

1: Yeah.

Moderator: So, I will reveal that we have had some focus groups with medical students, right, yes. And their views are different, but we can’t really—we can’t really reveal it, but I think depending on what your personal experience is and also depending on, like, what your interested in what you’re studying, that definitely reflected in your thoughts about that. Thank you so much—

2: Yeah. Thanks.

Moderator: --for participating, and for your time.

3: Thank you for having us.

1: Thank you very much.
